# A Review on the Catalytic Acetalization of Bio-renewable Glycerol to Fuel Additives

**DOI:** 10.3389/fchem.2018.00573

**Published:** 2018-11-26

**Authors:** Amin Talebian-Kiakalaieh, Nor Aishah Saidina Amin, Neda Najaafi, Sara Tarighi

**Affiliations:** ^1^Faculty of Petrochemicals, Iran Polymer and Petrochemical Institute (IPPI), Tehran, Iran; ^2^Chemical Reaction Engineering Group, Faculty of Chemical and Energy Engineering, Universiti Teknologi Malaysia (UTM), Skudai, Malaysia; ^3^Iran Industrial Design Company, Tehran, Iran

**Keywords:** glycerol, acetalization, fuel additives, heterogeneous catalysts, acetone, ketone

## Abstract

The last 20 years have seen an unprecedented breakthrough in the biodiesel industry worldwide leads to abundance of glycerol. Therefore, the economic utilization of glycerol to various value-added chemicals is vital for the sustainability of the biodiesel industry. One of the promising processes is acetalization of glycerol to acetals and ketals for applications as fuel additives. These products could be obtained by acid-catalyzed reaction of glycerol with aldehydes and ketones. Application of different supported heterogeneous catalysts such as zeolites, heteropoly acids, metal-based and acid-exchange resins have been evaluated comprehensively in this field. In this review, the glycerol acetalization has been reported, focusing on innovative and potential technologies for sustainable production of solketal. In addition, the impacts of various parameters such as application of different reactants, reaction temperature, water removal, utilization of crude-glycerol on catalytic activity in both batch and continuous processes are discussed. The outcomes of this research will therefore significantly improve the technology required in tomorrow's bio-refineries. This review provides spectacular opportunities for us to use such renewables and will consequently benefit the industry, environment and economy.

## Introduction

In the early Twentieth century, petroleum exploitation and its cracking to simple hydrocarbons was one of the most influential factors on human life. Fossil fuel has been the main source of energy for almost a century. Current oil production rate reach approximately 12 Mt/day and its demand is predicted to rise dramatically to around 16 Mt/day by 2030 due to the significant increase in the world population and industrial development (Lin and Huber, 2009; Talebian-Kiakalaieh et al., 2014). Various types of environmental concerns such as massive amount of carbon dioxide and the depletion of fossil fuel resources have become the main concerns in maintaining sustainability. Low cost supply of fossil fuel (<100 USD/barrel) will no longer be available by 2040 (Posada et al., 2009). Many efforts are geared toward finding new sources of alternative energy to supplant the current non-renewable fossil fuels. Biomass is selected as a promising alternative source of energy to meet the significant energy demand as well as to reduce environmental concerns. As a result, a new term, “bio-refinery,” has emerged recently to describe a facility for converting biomass to food, fuel, and value-added chemicals.

Biodiesel is one of the most important and valuable alternative liquid fuel in the transportation sector. As a substitute to fossil fuels, biodiesel could reduce chemical emissions such as sulfur dioxide (100%), unburned hydrocarbon (68%), and polycyclic aromatic hydrocarbon (80–90%). In addition, biodiesel is environmentally friendly, technically feasible and biodegradable (Fazal et al., 2011). The worldwide production of biodiesel is predicted to increase to 141 billion liters by 2022 from 110 billion liters in 2013, mainly due to the contribution of European Renewable Energy Directive (EU-RED) and Renewable Fuel Standard (RFS) in the United States. This would improve the global production to almost 70% by the year of 2022, compared to its average from 2010 to 2012 (SSI Review, 2014). Hence, it is vital to enhance the economic feasibility of biodiesel production through the modification of three major aspects of the process, namely the raw materials used in the process, the synthesis method, and the byproducts (De Torres et al., 2011).

Briefly, biodiesel is obtained via the transesterification of animal fat or vegetable oils in the presence of methanol under basic catalysis condition (Menchavez et al., 2017). Glycerol as a byproduct is produced at a high 1:10 glycerol to biodiesel weight ratio. The increasing demand for biodiesel caused a glycerol over-supply, thus reducing the commercial price of glycerol to almost 8 cents/lb recently compared to 25 cents/lb in 2004 (Clomburg and Gonzalez, 2013). It is expected the global surge in biodiesel production lead to production of >41.9 billion liter of crude glycerol by 2020 (Nanda et al., 2014a). Fabrication of low cost glycerol is important since it can be transformed to many value-added chemicals (more than 2,000 products) in various reaction pathways (Nanda et al., 2017; Nguyen et al., 2017; Tangestanifard and Ghaziaskar, 2017). Traditionally, glycerol was produced from the production of fatty acids (47%), followed by soaps (24%), fatty alcohols (12%), and the biodiesel industry (9%). However, since 2009 the biodiesel industry is the main producer that supplies over 64% of the glycerol (Abad and Turon, 2012). Thus, glycerol consumption is expected to increase significantly by up to 50% in 2020. Its demand was 2,247.2 kilo tons in 2013 and is expected to reach 3,469.2 kilo tons by 2020 (Ayoub and Abdullah, 2012; Villa et al., 2015).

Traditional uses of glycerol include in the textiles (24%), food and beverages (21%), cosmetics and toiletries (18%), drugs (18%), tobacco (6%), and paper and printing (5%), and others, cannot satisfy the dramatic surge in production of this compound. Thus, it is necessary to find new routes of conversion for this chemical in order to avoid market saturation. Table 1 lists the possible catalytic processes and products that can be produced from glycerol.

**Table 1:** Catalytic conversion of glycerol into value-added chemicals by different processes.

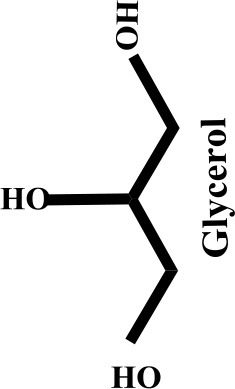	Oxidation	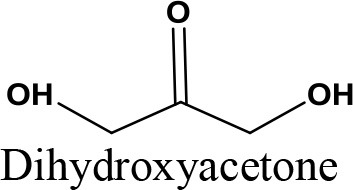	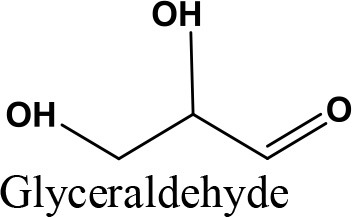	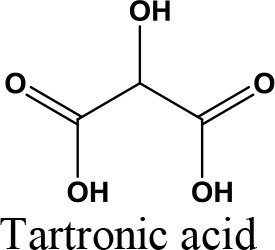	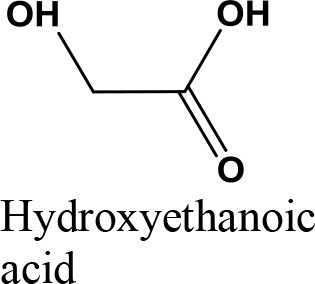	
		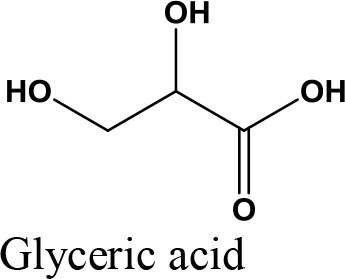	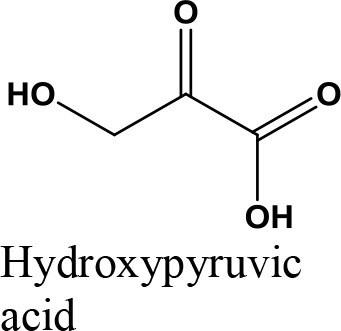	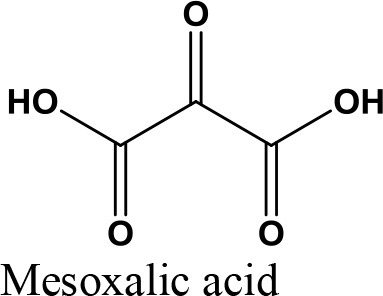	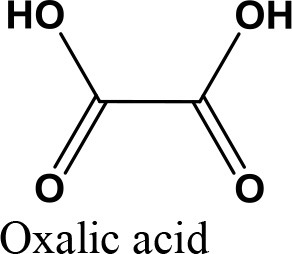	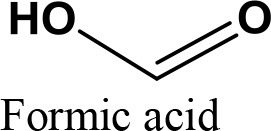
	Hydrogenolysis	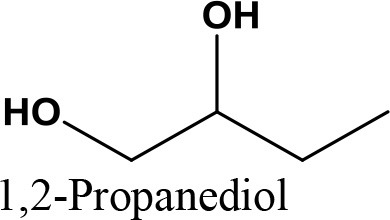	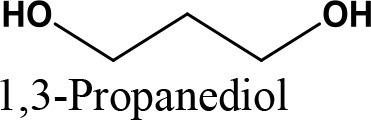	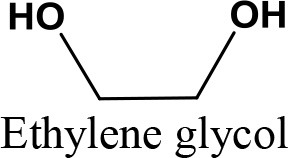	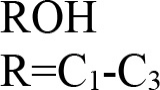	
	Dehydration	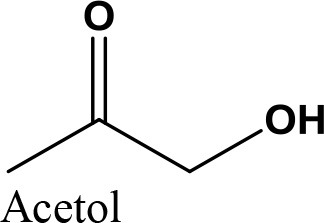	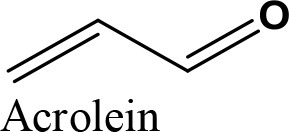			
	Pyrolysis, Gasification	C_n_H_2n+2_ Alkane	C_n_H_2n_ Olefin	ROH Alcohol	(CO + H_2_) Syngas	(C + H_2_) Carbon+ Hydrogen
	Trans- Esterification	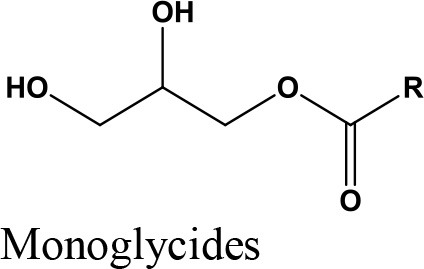		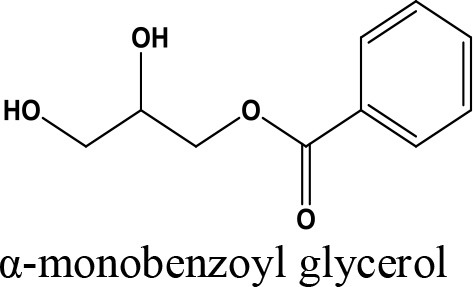	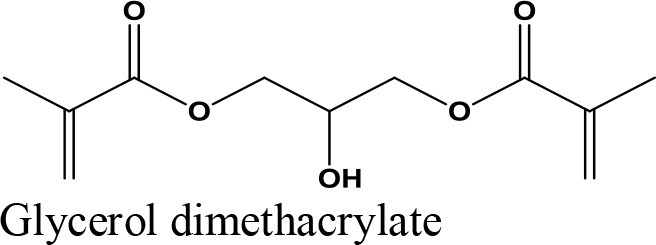	
	Etherification	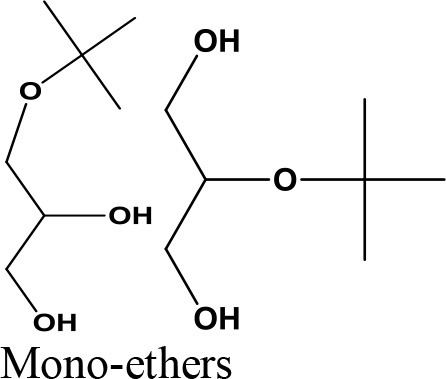		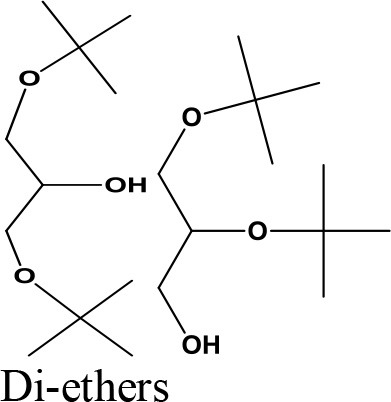	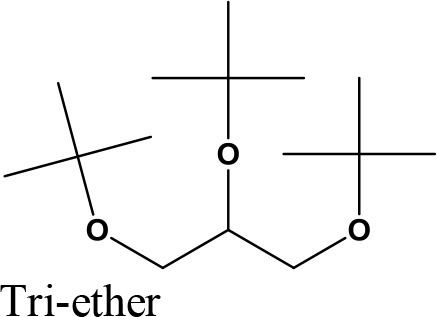	
	Oligomerization, Polymerization	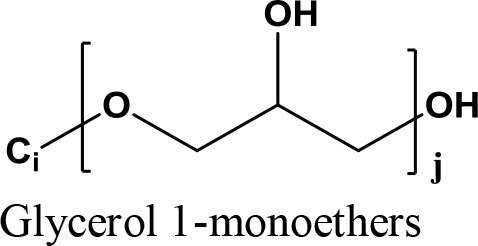		Polyglycerol methacrylates		
	Carboxylation	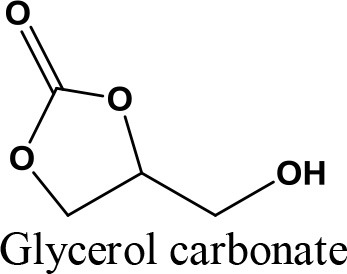				
	Acetalization	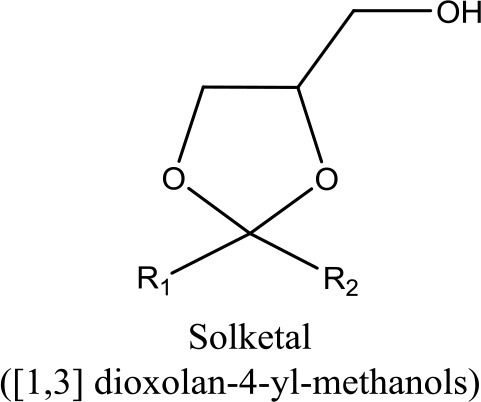		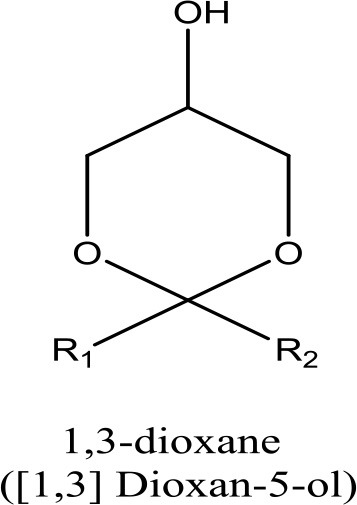		

*Different catalytic conversion of glycerol into value-added chemicals*.

**Figure 1:**
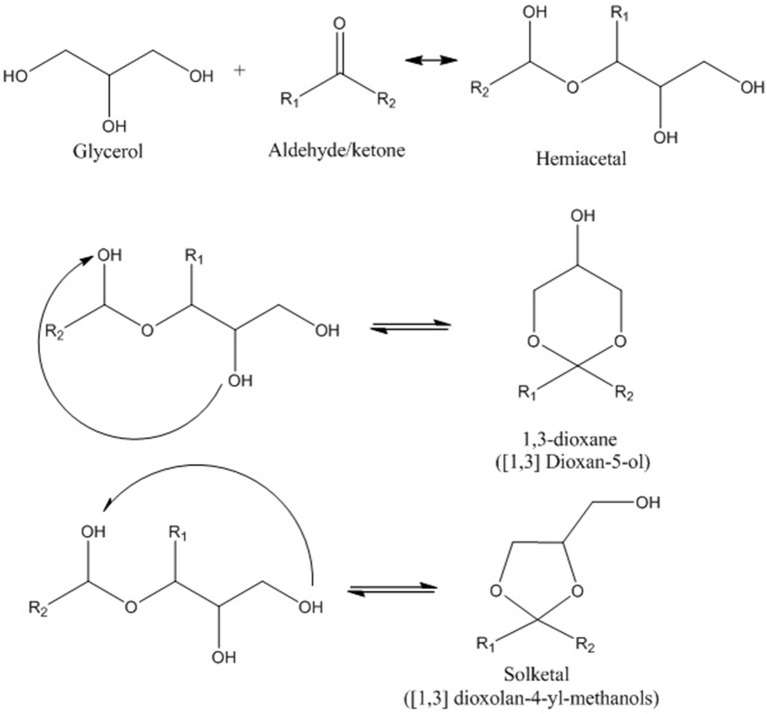
Reaction mechanism of glycerol with aldehydes/ketones.

Undoubtedly, one of the most promising glycerol applications is production of fuel additives such as cyclic acetals and ketals with aldehydes and ketones, respectively (Deutsch et al., 2007). Generally, fuel (Wang et al., 2004) and diesel additive (Ribeiro et al., 2007) is a material that improves the cleanliness of different parts of the engine (e.g., carburetor, fuel injector and intake valve), promotes complete combustion, reduces fuel gelling and choking of nozzle, as well as reducing corrosion impact on different parts of the engine. The result is improved engine performance, reduced emission and reduced fuel consumption. It could significantly reduce the particulate emissions of diesel fuel (Rakopoulos et al., 2008) (e.g., reduction of CO_2_ and NO emission) and increase oxygen and air concentration (Lin and Chen, 2006). In addition, it could improve the thermal stability of jet fuels as well as significantly reduce (1–70%) deposits in jet engines (Forester et al., 2003). Methyl tertiary butyl ether (MTBE) was widely used as octane accelerator in gasoline in the early 1980's (Franklin et al., 2000). For more than two decades, it was the most economical oxygenate additive used by the refineries to reduce production cost of Reformulated Gasoline (RFG) (Romanow, 1999). However, the International Agency of Research on Cancer (IARC) classified RFG as a major health risk threat in 2000 (U.S. EPA, 1996). Thus, ketalization reaction between glycerol and acetone where 2, 2-dimethyl-1, 3-dioxolane-4- methanol known as solketal, is formed as the condensation product over an acid catalyst is shown in Figure 1. Solketal, an oxygenate fuel additives, could reduce the particulate emission and improve the cold flow properties of liquid transportation fuels (Pariente et al., 2008). It helps to reduce the gum formation, improves the oxidation stability, and enhances the octane number when added to gasoline (Mota et al., 2010). Maksimov et al. (2011) reported its use as a versatile solvent and a plasticizer in the polymer industry and a solubilizing and suspending agent in pharmaceutical preparations. More importantly, the aquatox fish test on the toxicity of the solketal showed that solketal (with a LC50 for fish to be as high as 3,162 ppm) has demonstrated much less environmental toxicity than the common fuel additive, MTBE, with a LC50 of 1,000 ppm (Nanda et al., 2014a).

Thus, the main objective of this review is to collect information about the latest advances in glycerol conversion to oxygenated fuel additives from biomass sources. In addition, the review addresses the critical knowledge gaps for enhancing conversion and selectivity in glycerol acetalization. Fundamentals of reaction mechanisms for the acid-catalyzed conversion of glycerol into solketal are presented. Some aspects such as the influence of various reaction parameters, reactant selection, reaction temperature, catalyst acidity, water removal, and reactor design are exclusively summarized and discussed. Finally, the application of crude-glycerol is discussed in batch and continuous-flow processes.

## Catalytic acetalization of glycerol

Glycerol is an organic compound which is a low toxicity alcohol that consists of a three-carbon chain with a hydroxyl group attached to each carbon. These groups made glycerol hygroscopic and water-soluble. Glycerol has low volatility and low vapor pressure and is nontoxic to both humans and the environment. Physically, glycerol is a clear, colorless, odorless, viscous, and sweet-tasting liquid. Table 2 lists the physico-chemical characteristics of glycerol (Rahmat et al., 2010). Glycerol was first discovered by K.W. Scheele, a Swedish researcher, in 1779. He produced a material with a sweet taste by heating olive oil with lead oxide. Three decades later, a French chemist, Michel Eugene Chevrel, named it “glycerin.” He then proposed fatty acids ethereous chemical formulas along with glycerin formulas in vegetable oils and animal fats. Finally, his study on the production of fatty acids (FA) from the reaction of fatty materials with lime and alkali was reported, which was the first industrial attempt in this field (Gesslein, 1999). It is an irrefutable fact that the discovery of glycerol has brought significant breakthroughs in the production different products. Recent advances in catalyst and bio-refinery industries have provided great opportunities for industrialization of bio-based processes which could produce food, fuel, and chemicals from glycerol.

**Table 2:** Physico-chemical properties of glycerol.

**Properties**	**Values**	
Chemical formula	CH_2_OH–CHOH–CH_2_OH	
Formula weight	92.09	
Form and color	Colorless and liquid	
Specific gravity	1.260^50/4^	
Melting point	17.9°C	
Boiling point	290°C	
Solubility in 100 parts		
Water	Infinitely	
Alcohol	Infinitely	
Ether	Insoluble	
Vapor pressure in 760 mmHg	290°C	
Heat of fusion at 18.07°C	47.49 cal/g	
Viscosity liquid glycerol		
100%	10 cP	
50%	25 cP	
Diffusivity in	(DL × 10^5^ sq.cm/s)	
i-Amyl alcohol	0.12	
Ethanol	0.56	
Water	0.94	
**Specific heat glycerol in aqueous solution (mol%)**	**15**°**C (cal/g**°**C)**	**30**°**C(cal/g**°**C)**
2.12	0.961	0.960
4.66	0.929	0.924
11.5	0.851	0.841
22.7	0.765	0.758
43.9	0.670	0.672
100	0.555	0.576

Based on the literature in the last decades, catalytic acetalization of glycerol process could be categorized by three generations. In fact, the first generation of studies on the catalytic acetalization of glycerol to fuel additives reported in the presence of homogenous catalysts and a solvent. Fischer and co-workers pioneered the synthesis of solketal from glycerol and acetone, catalyzed by hydrogen chloride, in a batch reactor (Fischer, 1895). A few years later, a similar process by Fischer and Pfahler (1920) was applied for the ketalization of glycerol with anhydrous sodium sulfate and hydrogen chloride. Newman and Renoll (1945) reported the preparation of solketal in a three-neck flask using reflux and mechanical stirrer in 1948. To obtain high solketal yield, they used pTSA monohydrate as the catalyst and petroleum ether as the reaction medium. After the reaction, the products were separated by reducing the pressure and distilling. The drawback of this system was its very long reaction time (21–36 h). Generally, the reaction of glycerol with aldehydes/ketones is conducted under homogenous Lewis catalysts (Ruiz et al., 2010) or mineral acids such as HF, HCl, H_3_PO_4_, H_2_SO_4_, and p-toluenesulfonic acid (pTSA) to form solketal (1,2-isopropylidene glycerol, 2,2-dimethyl-1, or 3-dioxolane-4-methanol) (Sato et al., 2008; Coleman and Blankenship, 2010; Suriyapradilok and Kitiyanan, 2011; Nanda et al., 2014a; Sun et al., 2017). The first generation of studies was stopped more than half a century ago due to the economic barriers. Indeed, availability of cheap fossil fuels was the main obstacle for bio-based processes.

The second generation of catalytic acetalization of glycerol performed in the presence of heterogeneous catalysts and a solvent as reaction medium. Indeed, this group of investigations on bio-based glycerol acetalization to fuel additives started after the introduction of large amount of inexpensive glycerol from biodiesel industry at the end of the Twentieth century. Science and technological advances in synthesis of heterogeneous catalysts and their applications provide spectacular opportunities for further investigations in this field. In fact, homogenous catalysts like Lewis catalysts and strong mineral acids are known to not only cause difficult purification and product separation, but also environmental and corrosion problems. Several studies were employed to solve the shortcomings of homogenous catalysts using heterogeneous catalysts by evaluating the most important characteristics of a catalyst which are cost, accessibility, efficiency, easy removal, and good activity at mild conditions. One of the earliest studies on the use of heterogeneous acid catalyst was reported by Deutsch et al. (2007). They applied Amberlyst-36 with various solvents (dichloromethane, chloroform, toluene, and benzene) as organic solvents to obtained >62% glycerol conversion in the presence of three different reactants (acetone, benzene, and furfural) in a batch reactor (Deutsch et al., 2007). Application of Hβ and MMT-K10 zeolites were reported in catalytic acetalization of glycerol to fuel additives in the presence of chloroform as solvent and benzaldehyde as reactant. The results indicated that >95% of solketal yield was obtained at glycerol to benzaldhyde molar ratio of 1.1/1 and after 6 h of reaction time (Deutsch et al., 2007). In addition, toluene is another solvent which was utilized in catalytic acetalization of glycerol by Umbarkar et al. (2009). They reported about 72% glycerol conversion over MoO_3_/SiO_2_ catalyst at optimum reaction of 1.1/1 molar ration of glycerol to benzaldehyde, reaction temperature of 100°C and in 8 h. As mentioned earlier, simultaneous application of heterogeneous catalysts and solvent is one of the old methods in catalytic acetalization process and there are limited number of studies in this field in the last decade. However, Nanda et al. (2014b) reported one of the successful studies on application of ethanol as solvent in the presence of Amberlyst-35 as catalyst to reach more than 74% solketal yield at 2/1 molar ratio of glycerol to acetone and quite very low reaction temperature of 25–45°C. Indeed, they could significantly reduce the reaction temperature by application of ethanol as solvent.

Finally, the third generation is solvent free glycerol acetalization reaction by heterogeneous catalysts in batch or continuous processes. In fact, new heterogeneous catalysts are active enough to push the catalytic process to produce desired products (solketal) at high reaction conversion even without solvent (Chen et al., 2018a; Ferreira et al., 2018). In this regard, different types of heterogeneous acid catalysts have been recently applied in the acetalization of various carbonyl compounds with glycerol such as activated carbons, montmorillonite (MMT), zeolites, metal-based catalysts, ionic liquids, supported multi-walled carbon nano-tubes (MWCNTs) or, mesoporous silicates with arylsulphonate group, heteropoly acids, rare-earth triflates, and ion-exchange resins. Thus, the latest trend in catalytic acetalization of glycerol to fuel-additives will be investigated in the following sections. Table 3 summarizes some of the recent studies related to glycerol acetalization with different aldehydes and ketones in batch and continuous processes. All the reported studies are organized into two main groups of homogenous and heterogeneous catalysts. Also, the heterogeneous catalysts are divided into four categories of zeolite-, heteropoly acid-, metal-, and polymers-based catalysts. As it can clearly be seen, the highest catalytic activity (complete conversions) were observed from the rare-earth triflate catalysts (Pierpont et al., 2015), Ni-Zr/activated carbon catalyst (Khayoon and Hameed, 2013), (L)Ru (II)@SBA-15 (Lazar et al., 2018), Amberlyst-47 (Guemez et al., 2013), and [5%V] Si-ITQ-6 (Vieira et al., 2018). The detail of each reaction process and optimum conditions reported in Table 3.

**Table 3:** Glycerol acetalization with different aldehydes and ketones in batch and continuous processes.

**Type**		**Catalyst**	**Optimum condition**	**Con^c^ (%) Y^d^ (%)**	**Description**	**References**
Homogenous		PTSA	MR^a^Gl/F^b^ = 1:1T = 80°C, *t* = 9 h	Y_Acetal_ = 80	–	Ruiz et al., 2010
		PTSA	MR Gl/Ac^e^ = 1:4*t* = 12 h	Con = 82	–	Suriyapradilok and Kitiyanan, 2011
		PTSA	MR Gl/Ben^f^ = 1:2*T* = 140°C, *t* = 15 min	Con = 67	Microwave assisted, Power = 600 W; Con = 95% without catalyst	Pawar et al., 2014
		H_2_SO_4_	MR Gl/F = 1.5/1*T* = 100°C, *t* = 4 h	Y_Acetal_ = 89	–	Coleman and Blankenship, 2010
Heterogeneous	Zeolites	Zeolite beta	MR Gl/F = 1/1 *T* = 100°C, *t* = 2 h	Y_Acetal_ = 25	–	Ruiz et al., 2010
		Zeolite beta	MR Gl/Ac = 1/2 *T* = 70°C, *t* = 1 h	Con = 90	–	da Silva and Mota, 2011
		H beta zeolite	MR Gl/Ac = 1/2 *T* = 25°C, *t* = 2 h	Con = 86	–	Manjunathan et al., 2015
		Zeolite USY	MR Gl/Bu^g^ = 1/2.5 *T* = 70°C, *t* = 4 h	Con = 72	–	Serafim et al., 2011
		Zeolite BEA		Con = 87		
		Zeolite ZSM		Con = 28		
		MMT K10	MR Gl/Ben = 1/2 *T* = 140°C, *t* = 15 min	Con = 84	Microwave assisted, Power = 600 W	Roldan et al., 2009
				Con = 95	No catalyst	
		Nb_5_-HUSY	MR Gl/Ac = $\frac{1}{2}$ *T* = 40°C, Cat = 2 wt%	Con = 66S_Solketal_ = 98	–	Ferreira et al., 2018
		MK-10S_MW_	*T* = 40°C, *t* = 2 h, Cat = 5 wt%, MR Gl/F = 1/1	Con = 68S_Solketal_ = 66	Microwave synthesis enhanced reaction conversion	Gutiérrez-Acebo et al., 2018
		[5%V]Si-ITQ-6	*T* = 60°C, *t* = 120 min, Ac/Gl = 3/1, Cat = 0.02 g	Con = 100S_Solketal_ = >95	Acetone washing could reduce the catalyst deactivation after each run	Vieira et al., 2018
		Zr-MO-KIT-6	*T* = 50°C, *t* = 4 h, Ac/Gl = 8/1, Cat = 0.05 g	Con = 85.8S_Solketal_ = 97.8	–	Li et al., 2018a
		Immobilize sulfonic acid on to silica	*T* = 120°C, *t* = 8 h	Con = 78	Argon atmosphere in the presence of Benzaldehyde	Adam et al., 2012
		Zeolite betaCP814E	MR Gl/Ac = 1/6 *T* = 35°C, *t* = 4 h	Con = 82%	–	Maksimov et al., 2011
		Zeolite betaCP811T1		Con = 85%		
		Zeolite HY		Con = 37%		
Heterogeneous	Zeolites	Hierarchical Zeolite (H/BEA_5_)	*T* = 70°C, *t* = 240 min, MR G/F = 1/1.25, Cat = 10%	Con = 78S_Solketal_ = 85	–	Sonar et al., 2018
		6.8v-MCM-41	*T* = 60 °C, *t* = 60 min, Ac/Gl = 6.5, Cat = 20 mg	Con = 92S_Solketal_ = 95	–	Abreu et al., 2018
		ITQ-2	T = 83°C, HMF/Gl = 1/2, Cat = 20 wt%, Si/Al = 15	Con = 98S_5R+6R_ = 100	Products ratio 5R/6R = 2.8	Arias et al., 2018
		MCM-41		Con = 99S_5R+6R_ = 100	Products ratio 5R/6R = 3.9	
	Heteropoly acid	Cs_2.5_H_0.5_PW_12_O_40_	*T* = 25°C, MR Gl/Ac = 1/6, Cat = 0.25 g/batch, *t* = 15 min	Con = 95S_Solketal_ = 98	–	Chen et al., 2018a
		Cs_2.5_/KIT-6	*T* = 70°C, MR Gl/F = 1/1.2, C_s_ = 3.83 g/batch, *t* = 24 h	Con = 95Y_GF_ = 60	–	Chen et al., 2018b
	Acid exchange resins	Nafion SAC 13	MR Gl/Ben = 1/2 *T* = 140°C, *t* = 15 min	Con = 81	Microwave assisted, Power = 600 W, Con = 95% without catalyst	Trifoi et al., 2016
		Dowex	MR Gl/Bu = 1/2.5 T = 70°C, *t* = 4 h	Con = 66	–	Serafim et al., 2011
		Amberlyst 36	MR Gl/F = 1/1, *T* = 100°C, *t* = 4 h	Y = 55	–	Ruiz et al., 2010
		Amberlyst 15	MR Gl/Ac = 1/2 *T* = 70°C, *t* = 1 h	Con = 95	–	
		Amberlyst 15	MR Gl/Ac = 1/2 *T* = 50°C, *P* = 8.0 bar *t* = 6 h	Con = 95	–	
		Amberlyst 47	MR Gl/F = 2/1 *T* = 80–100°C, *t* = 3 h	Con = 75	–	Agirre et al., 2011
		Amberlyst 47	MR Gl/Ac = 2/1 *t* = 10–50°C, *t* = 4 h	Con = 90		
		Amberlyst 47	MR Gl/But^h^ = 3/1 *T* = 80°C, *t* = 100 min	Con = 95	–	Guemez et al., 2013
			MR Gl/But = 0.5/1 *T* = 60 °C, t = 4 h	Con = 100		
		Amberlyst 15	MR Gl/F = 1/2 T = 75°C, *t* = 2 h	Con = 100%	Reactive distillation process	Hasabnis and Mahajani, 2014
Heterogeneous	Metal-based	Ni-activated carbon	MR Gl/Ac = 1/8 *T* = 45°C, *t* = 3 h	Con = 98	3% reduction of catalytic activity after the 4th run	Khayoon and Hameed, 2013
		Zr-activated carbon		Con = 67		
		X%Ni-Y%Zr/ activated carbon		Con = 100		
		Ni-MWCNT ^i^	MR Gl/Ac = 1/6 *T* = 40°C, *t* = 3 h	Con = 96	5% reduction of catalytic activity after 4th run	Khayoon et al., 2014
		Pt-TNT	*T* = 50°C, *t* = 24 h, Ac/Gl = 1/1, Cat = 130 mg	Con = 46.7S_Solketal_ = 10	–	Gomes et al., 2018
		M-AlPO_4_M-ZnAlPO_4_M-CuAlPO_4_M-NiAlPO_4_M-CoAlPO_4_	MR Gl/Ac = 1/8 *T* = 80°C, *t* = 1 h	Con = 75	80% reduction of M-NiAlPO_4_ activity after the 5th run	Zhang et al., 2015
		PTNT	*T* = 50 °C, MR G/Ac = 1/1, *t* = 6 h	Con = 40S_Soketal_ = 20	–	Gomes et al., 2018
		SO_4_/SnO_2_	Gl-Fur^j^	Con = 99	–	Mallesham et al., 2014
		TiO_2_-SiO_2_	MR Gl/Ac = 1/4 *T* = 40–90°C, *t* = 3 h	Con = 98	${\text{Sel}}_{5-\text{memberedring}}^{\text{k}}$ = 95%	Fan et al., 2012
		Mo_X_/TiO_2_-ZrO_2_	MR Gl/Ben = 1/1 *T* = 60–100°C, *t* = 90 min	Con = 74	–	Sudarsanam et al., 2013
		Niobium oxyhydroxyde	MR Gl/Ac = 1/4 *T* = 40°C, *t* = 1 h	Con = 74	–	Souza et al., 2014, 2015
		Nb_2_O_5_	MR Gl/Ac = 1/3 T = 70 °C, t = 6 h	Con = 80	Up to 4 time reusability	Nair et al., 2012
		HC-SZ (${\text{SO}}_{4}^{2}$/ZrO_2_ coated on cordierite honeycomb monolith)	*T* = 60°C, MR G/Ac = 1/3, Cat = 0.2 g	Con = 96, Y_Solketal_ = 94	–	Vasantha et al., 2018
		Meso-SnO_2_-350	*T* = 60°C, *t* = 30 min, Ac/Gl = 1/1, Cat = 0.125 g	Con = 51.3S_Solketal_ = 98	Higher selectivity to the solketal in the presence of Acetone compared to the Furfuraldehyde and benzaldehyde	Manjunathan et al., 2018
Heterogeneous	Other catalysts	Rare earth triflate	Gl-Ac *T* = 25 °C	Con = 100	–	Pierpont et al., 2015
		Organic-inorganic hybrid catalyst	MR Gl/Ac = 1/6 *T* = 30°C, *t* = 3 h	Con = 94	Water resistance	Sandesh et al., 2015
		(L)Ru(II)@SBA-15	*T* = 25°C, *t* = 20 min, MR Al/MeOH = 1/250	Con = 100S_Solketal_ = 100	–	Lazar et al., 2018
		80LS20PS450H^+^	*T* = 40°C, Cat = 5 wt%, MR G/Ac = 1/6, *t* = 60 min	Con = 90	S_Soketal_ = 51–53% obtained over Furfural and Methyl levulinate instead of acetone	Konwar et al., 2017
		PrSO_3_H-SBA-15-400	*T* = 90°C, *t* = 8 h, F/Gl = 1.5/1, Cat = 0.2 g	S_Solektal_ = 60	–	Li et al., 2018b
		Carbon-based catalyst	*T* = 28°C, *t* = 30 min, Ac/Gl = 4/1, Cat = 3 wt%	Con = >78S_Solketal_ = 73	–	Mantovani et al., 2018
		Co(II)!!!!(Co(III)_1.25_)Al_2−0.75_)O_4_	*T* = 130°C, *t* = 3 h, 2 g Gl and 12.72 g AC, Cat = 0.1 g	Con = 69.2S_Solketal_ = 98.6	–	Li et al., 2018c
		Purolite PD206	*T* = 40.66°C, *P* = 42.31 bar, MR Gl/Ac = 1/4.97, Feed flow rate = 0.49 ml/min, Cat = 0.5 g	Normalized exergy destraction = 6.18%, Universal Exergetic efficiency = 90.36%	Optimization and modeling of continuous acetalization process with subcritical acetone	Aghbashloa et al., 2018
		KU-2	MR Gl/Ac = 1/6 *T* = 60°C, *t* = 4 h	Con = 85%	–	Maksimov et al., 2011
		Purolite PD 206	MR Gl/Ac = 5/1 *T* = 20°C, *P* = 120 bar	Con = 95%	Acetone-solvent	Shirani et al., 2014

a MR, Molar Ratio;

b F, Formaldehyde;

c Con, Conversion (%);

d Y, Yield (%);

e Ac, Acetone;

f Ben, Benzaldehyde;

g Bu, Butanal;

h But, Butiraldehyde;

i MWCNTs, Multiwall carbon nano-tubes;

j Fur, Furfural;

^k^Sel, Selectivity.

Lack of research studies on different methods of catalyst synthesize (e.g., sol-immobilization) is obvious, which has great influence on the catalyst activity and selectivity. In fact, the majority of reported heterogeneous catalysts synthesized through simple impregnation approach. Also, the photo-catalytic process is a promising strategy in various reaction processes and under mild reaction conditions with altered selectivities, compared with the conventional thermo-catalytic route. Unfortunately, application of photo-catalytic acetalization of glycerol has rarely been reported, while it definitively requires more attention in the future.

**Figure 2:**
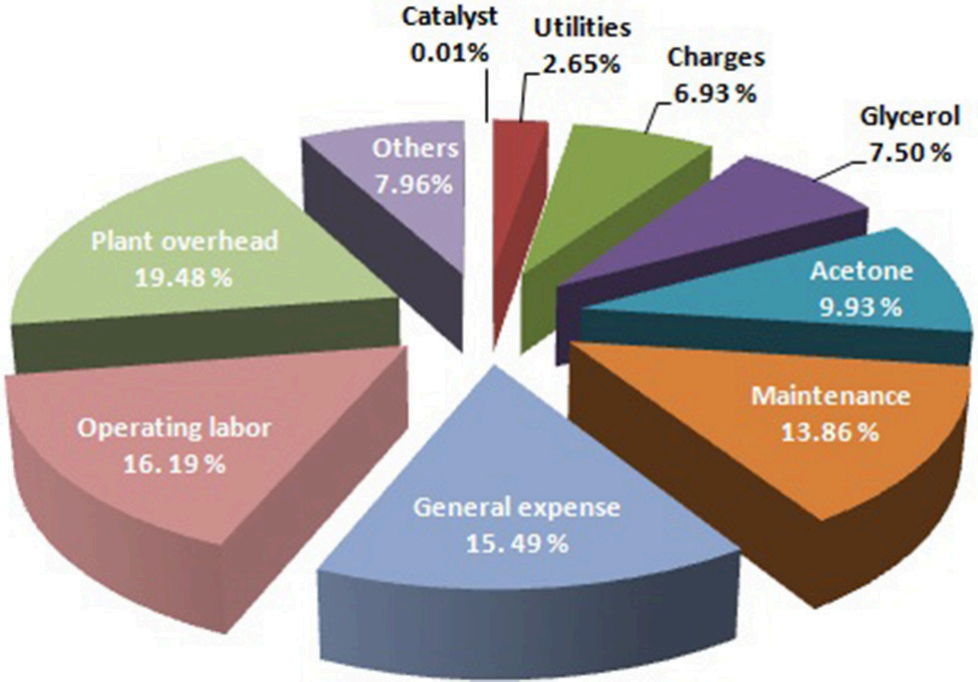
Annual operation costs.

In addition to investigating the synthesis of an effective catalyst, the process engineering for economic evaluation was also rarely investigated. The UNISim^TM^ software was used for the material and energy balances. The proposed plant could consume 432 t/y of glycerol and produce 620.9 t/y of solketal. The solketal cost was 12.29US$/kg. The annual operation costs are shown in Figure 2. The results of this study suggest that glycerol acetalization for the production of solketal (fuel-additive) requires more attention and study. Such simulation studies could provide valuable information before large-scale industrialization of glycerol acetalization process. Indeed, researchers could analyses and evaluate different scenarios to evaluate the environmental and economic aspects of this process such as material, energy and production cost.

### Zeolite based (micro- and mesoporous) catalysts

Zeolite is a micro-porous, alumino-silicate mineral conventionally used as commercial adsorbents due to its unique porous characteristics (tunable pore size), acid sites, and high thermal stability (Halgeri and Das, 1999). Zeolites could be used in various applications with a global market of several million tons annually, which includes petrochemical, water purification, gas separator, nuclear, and biogas industries. Among different forms of zeolite, nano-crystalline zeolite Beta and Y showed higher activity than micro-crystalline zeolites due to their high surface area, lower diffusion path length and more exposed active sites (Taufiqurrahmi et al., 2011).

**Figure 3:**
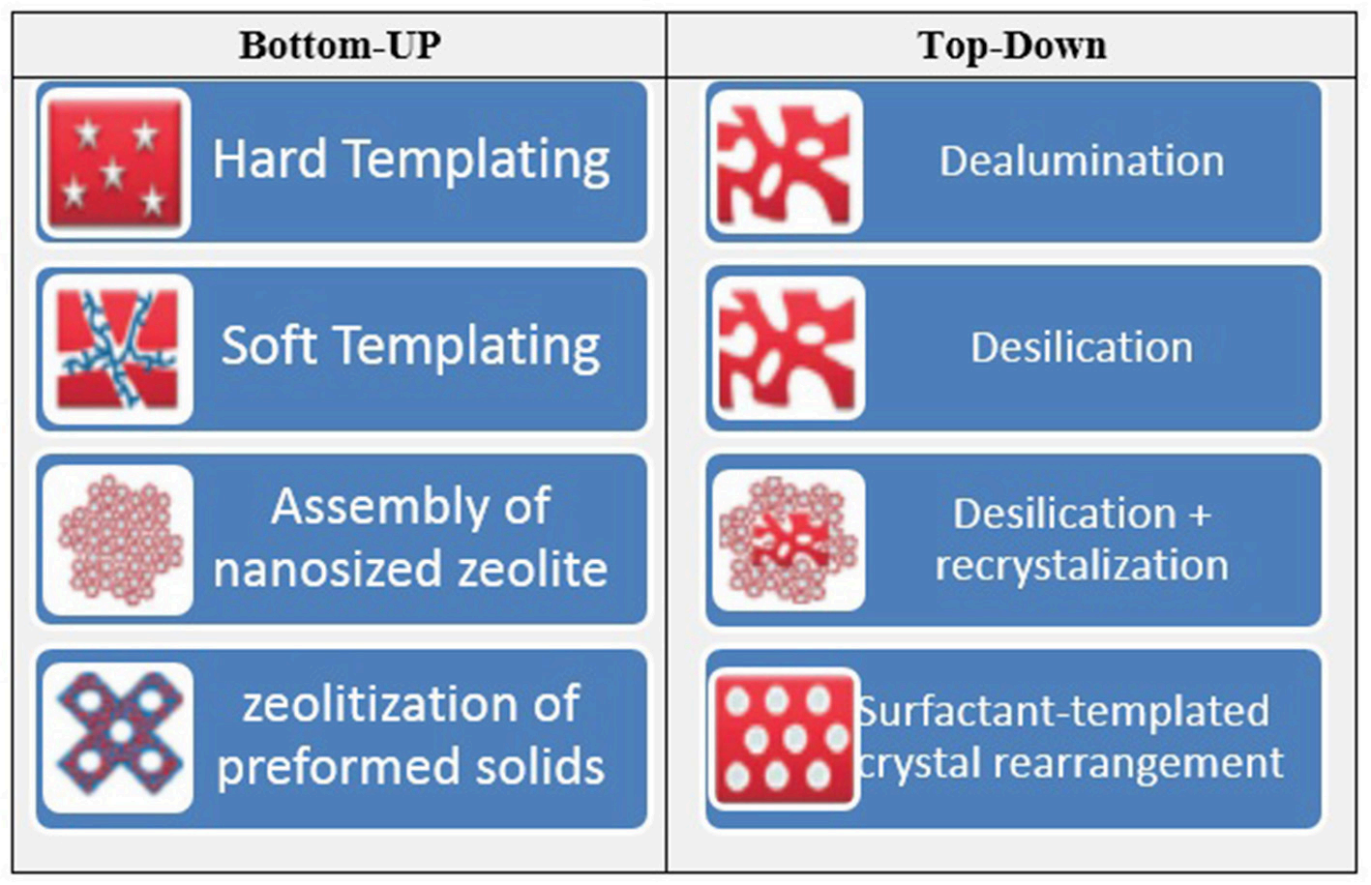
Bottom-up and top-down models for synthesizing hierarchical mesoporous zeolites.

Despite all the research studies which have been used zeolite catalysts, some studies reported diffusion problems (mass transfer resistance) by utilization of bulk zeolites due to the presence of micro-porous network (Sharma et al., 2011). Thus, researchers have decided to use different metal oxides, metals, or metal nanoparticles as a support for zeolites to overcome these limitations. However, the synthesis procedures are sometimes laborious and require additional costs due to application of noble metals or thermal treatment which in general consume lots of time, energy, and cost. As a result, new concept of “Hierarchical zeolites” have attracted much attention recently. Based on our knowledge, application of supported hierarchical zeolites acid catalysts is reported rarely in this field. Indeed, hierarchical zeolites overcome the drawbacks related to hamper mass transfer and limited accessibility of conventional zeolites by the introduction of secondary, larger porosity within the micro-porous framework (García-Martínez and Li, 2015). In hierarchical meso-micro-porous zeolites, mesopores facilitate the physical transport of reactant molecules, whereas micropores act as nano-reactors to provide both active sites and shape selectivity (Groen et al., 2007). Therefore, hierarchical zeolites have recently been explored as catalysts for reactions that involve bulky molecules and their outstanding activities have been reported (Zhou et al., 2010). There are two approaches to introduce a hierarchical pore structure (connected pore structure) in zeolites. In fact, the bottom up and the top-down methods which hierarchical zeolites are synthesized directly from a silica-alumina gel or by post-treatment of the existing zeolites, respectively. Extra-crystalline, hard, templates such as carbon, (Egeblad et al., 2008) starch, (Park et al., 2009) resins, (Tosheva et al., 2000) and surfactants, (Choi et al., 2006) which are removed by calcination after crystallization to create mesoporosity can be used in the bottom-up approach (Fan et al., 2008). In the top-down approach to achieve hierarchical form, zeolites are post-treated after synthesis. The easiest way to introduce mesoporosity is by dealumination, which can be achieved by steaming and chemical treatments, such as acid leaching which remove the resulting extra-framework alumina. The increased mesoporosity may give rise to increasing rates in bimolecular and oligomeric reaction pathways that require large transition states (Lupulescu and Rimer, 2012). Another way of producing mesopores is desilication which can be done by base leaching. Figure 3 illustrates bottom-up and top-down methods for synthesizing hierarchical meso-porous zeolites (Vogt and Weckhuysen, 2015). Undoubtedly, application of hierarchical zeolites as one of the catalysts with high activity and selectivity to the desired product should be more studied in the glycerol acetalization process due to its characteristics and acceptable results in other chemical processes particularly as a fluid catalytic cracking (FCC) catalyst in petrochemical industry.

One of the early studies regarding application of zeolites, was performed by da Silva et al. (2009) who investigated different catalysts (K10 MMT, zeolite Beta, amberlyst 15, and p-toluene sulfonic acid) for the conversion of glycerol to fuel-additives in the presence of acetone or formaldehyde. Consequently, the zeolite Beta (Si/Al = 16) reached conversion >95% in 1 h. In fact, high content of Si/Al ratio led to the hydrophobic characteristic of zeolite, which prevents the diffusion of water to inside the pores and acid sites' strength was preserved. Nevertheless, with aqueous formaldehyde solution, the glycerol conversion illustrated a drop to between 60 and 80% for different catalysts (Amberlyst-15, K-10 montmorillonite, p-toluene-sulfonic acid). Indeed the main reason was high amount of water in the reaction medium, which shifts the equilibrium and weakens the acid sites.

**Figure 4:**
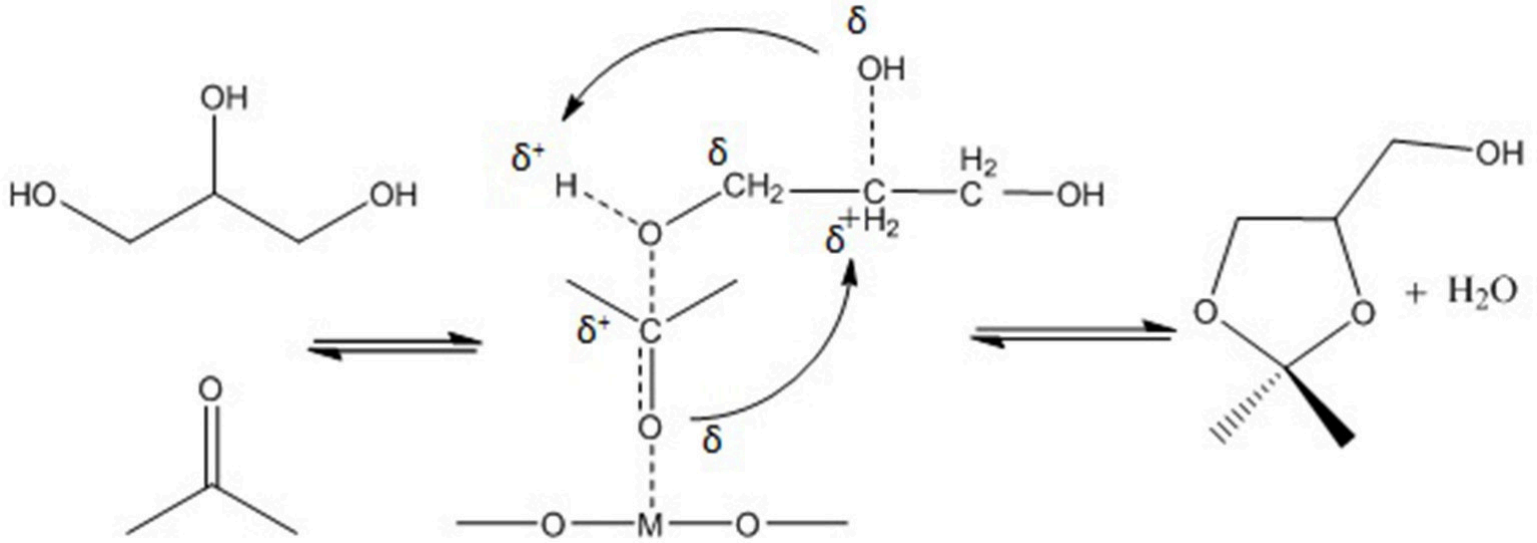
Proposed reaction mechanism for the acetalization of glycerol and acetone over Lewis acid catalyst.

Li et al. (2012) reported that mesoporous Lewis acid catalysts could be active in acetalization of glycerol with acetone to produce solketal. A series of three-dimensional mesoporous silicate catalysts (Hf-TUD-1, Zr-TUD-1, Al-TUD-1, and Sn-MCM-41) synthesized and two of these catalysts (Hf-TUD-1 and Zr-TUD-1) showed excellent catalytic activities in solketal production. Indeed, 65 and 64% glycerol conversion obtained over Hf-TUD-1 and Zr-TUD-1 catalysts, respectively, at optimum reaction condition of 2/1 molar ratio of acetone to glycerol, 25 mg of catalyst weigh, at 80°C in 6 h reaction time. The main reasons for such high activity of synthesized catalysts were wide pores (Hf-TUD-1 = 0.6 cm^2^/g, Zr-TUD-1 = 0.8 cm^2^/g), large specific surface area (Hf-TUD-1 = 715 m^2^/g, Zr-TUD-1 = 651 m^2^/g), large pore size (Hf-TUD-1 = 4 nm, Zr-TUD-1 = 13.3 nm), the amount of accessible acid sites, and a relatively hydrophobic surface of catalyst. In addition, the active mesoporous materials didn't suffer from leaching and could be efficiently reused in consecutive catalytic cycles. They also proposed a reaction mechanism for acetalization reaction in the presence of Lewis acid catalysts. The Lewis acid metal sites coordinate and activate acetone's carbonyl group. Then, the carbon atom of the carbonyl group is attacked by the primary alcoholic group of glycerol accompanied by the formation of a bond between the carbonyl oxygen atom and the secondary carbon atom of glycerol. Finally, solketal forms through the dehydration step. Figure 4 displays the detailed reaction mechanism.

Jamil et al. (2017) used different tailored forms of zeolite Beta in the condensation of bio-glycerol with acetone for production of the Solketal. The zeolite Beta catalysts treated with acids (hydrochloric acid, nitric acid, and oxalic acid) exhibited enhanced catalytic activity, irrespective of the nature of the acid used for the de-alumination. The nitric acid-treated beta zeolite sample (AB-2) exhibited a higher conversion than the other acid-treated samples. At optimum conditions (1:6 glycerol to acetone molar ratio, 4 h reaction time, 60°C reaction temperature) the bio-glycerol conversion and solketal yield were 94.26% and 94.21 wt%, respectively. The AB-2 sample was reusable for at least 4 times without any significant loss in its activity with approximately >80% glycerol conversion and >80% solketal yield.

Kowalska-Kus et al. (2017) investigated the glycerol acetalization reaction with acetone in the presence of hierarchical zeolites comprising pores of different diameters (MFI, BEA, and MOR) at 343 K and 1:1 glycerol to acetone molar ratio. The best catalytic performance for glycerol acetalization, which was 100% solketal selectivity at 80% reaction conversion, was achieved over hierarchical (micro/mesoporous) MFI zeolites. A significant increase in reaction conversion and solketal selectivity in the studied reaction resulted from the easier accessibility of the active sites to reagents due to the formation of mesopores by means of desilication of the micro-porous zeolites.

### Heteropoly acid based catalysts

**Figure 5:**
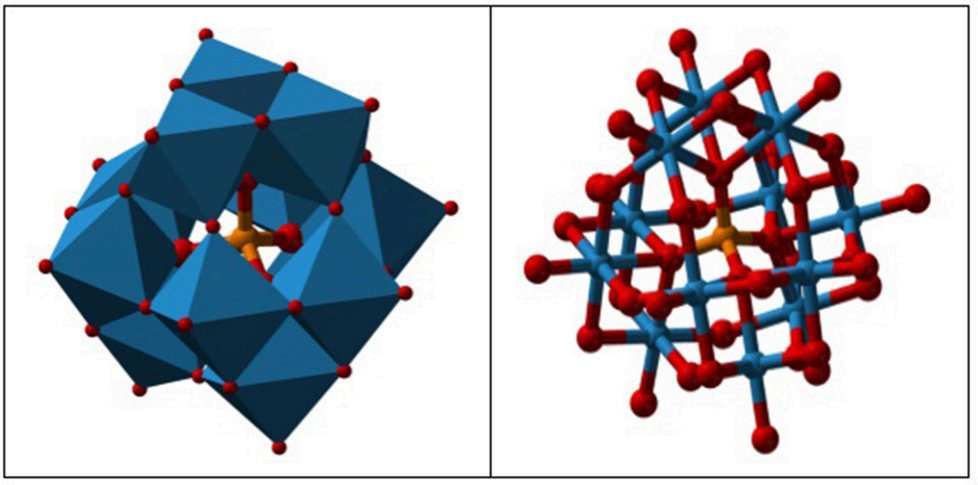
HPA keggin structure.

Application of heteropoly acid (HPA) has attracted much attention due to its wide applications in biodiesel industry and production of value-added chemical from glycerol. HPAs are highly stable against humidity and air, low toxicity, high solubility in polar solvents, production of less residues than mineral acids, less corrosive, and highly safer than other catalysts (Martin et al., 2012). Tungstophosphoric acid (HPW), silicotungstic acid (HSiW), and Phosphomolybdic acid (HPMo) are three commercially available HPAs. The HPW is a common HPA catalyst which is widely used. HPA catalysts have high ability for adjustment by modifying their central atoms with various compounds. Researchers have attempted to increase the catalytic activity and long-life stability of the catalysts to achieve the highest fuel-additive yield. The Cs/HPW catalyst displayed one of the highest potential catalyst in acetalization of glycerol with 98% selectivity to solketal at about 95% glycerol conversion (Chen et al., 2018a). HPA's possessed Keggin structure. It is the structural form of α-Keggin anions, which have a general formula of [XM_12_O_40_]^n−^, where X, M, and O represent the heteroatom, the addenda atom, and oxygen, respectively (Figure 5). The structure self-assembles in acidic aqueous solution and is the most stable structure of polyoxometalate catalysts. Despite the enormous applications of HPA catalysts as active components in various heterogeneous catalytic processes [e.g., glycerol dehydration to acrolein (Talebian-Kiakalaieh et al., 2014), glycerol oxidation to glyceric acid (Talebian-Kiakalaieh et al., 2018)], application of these types of catalysts are rarely reported in glycerol acetalization reaction.

**Figure 6:**
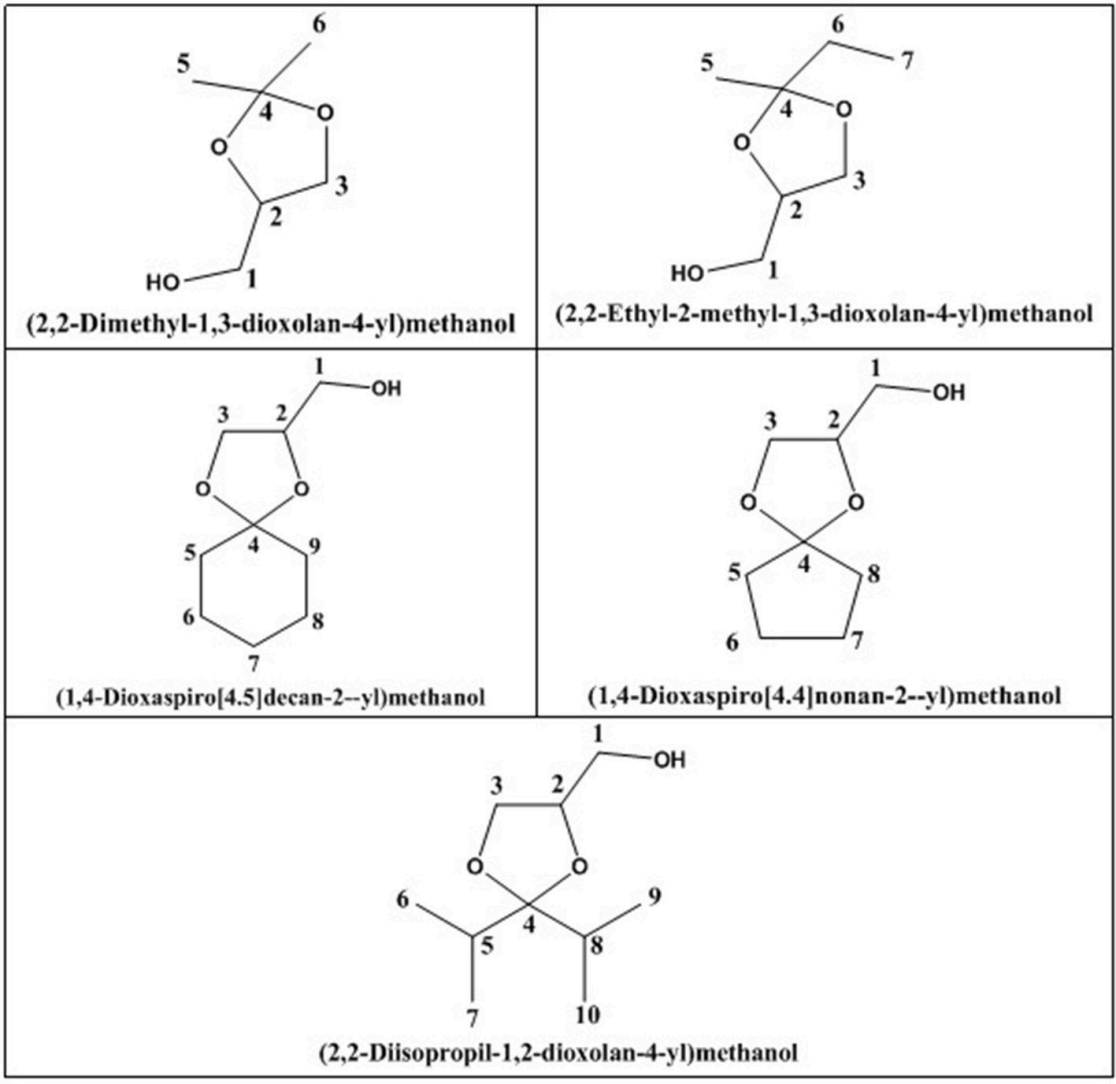
Products with application of various ketones.

The glycerol acetalization was studied using a series of supported HPAs [HPW, HPMo, HSiW, and molybdosilisic (SiMo)], immobilized in silica catalysts by sol–gel method (Ferreira et al., 2010). As results, all catalysts exhibited high solketal selectivities (near S_Solketal_ = 98%) at quite complete conversions at optimum reaction conditions of 70°C reaction temperature, 0.2 g catalyst weight, 6:1 molar ratio of acetone to glycerol and after 4 h reaction time. Also, the catalytic activities decreased in the following order: HPW-S > SiW-S >PMo-S > SiMo-S. All the catalysts exhibited high stability even after the fourth consecutive run, having lost only 10–13% of their initial activity. In another study, Narkhede and Patel (2014) achieved high selectivity toward solketal using supported SiW with MCM-41 catalysts (30% SiW_11_/MCM-41, 30% SiW_12_/MCM-41) in the presence of benzaldehyde. The results indicated that the 30%-SiW_11_/MCM-41 could reach the highest solketal selectivity of 82 at 85% glycerol conversion at room temperature (30°C), 1/1.2 molar ratio of glycerol to benzaldehyde, 100 mg catalyst weight and in 1 h. Also, tuning of the acidity of the parent SiW led to an increase in the selectivity toward solketal. High activity of these catalysts was attributed to their strength of acidity, wide pores and large specific surface area.

da Silva et al. (2015b) evaluated the activity of various Brønsted acid catalysts e.g., HPW, H_2_SO_4_, p-toluene sulfonic acid, PMo, or SiW on glycerol ketalization with different ketones (e.g., propanone, butanone, cyclopentanone, and cyclohexanone) at room temperature and in the absence of an auxiliary solvent. The HPW sample exhibited the highest activity among the Brønsted acid catalysts and exhibited high (> 85%) selectivity toward five-membered (solketal) cyclic ketals. The highest (98%) selectivity of solketal is obtained at 288 K reaction temperature, 1 mol% catalyst (HPW) loading, 1:30 glycerol to ketone (propanone) molar ratio. The activity of different tested catalysts was as follows HPW> *p*-toluene sulfonic acid > PMo > SiW > H_2_SO_4_ with 83% > 76% > 41% > 40% > 31%, respectively. In addition, the results revealed that the application of various ketones with Brønsted acid catalyst in absence of solvent for ketalization of glycerol has significant influence on product distribution. Figure 6 summarizes the possible products that can be obtained as a result of different ketone application.

### Metal based catalysts

Mixed oxides, phosphates, and pyrophosphates have been used in glycerol acetalization to fuel-additives. Metal oxide catalysts such as niobium oxide (Nb_2_O_5_), tungsten oxides (WO_3_), silicon dioxide (SiO_2_) have been widely used in various chemical processes. The most important factors about metal-based catalysts are their synthesis method (especially calcination temperature) and their binary or tertiary combinations which have detrimental impact on physicochemical characteristics catalyst (Talebian-Kiakalaieh et al., 2014).

Mallesham et al. (2013) synthesized a series of supported SnO_2_ with molybdenum (Mo) and tungsten (W) solid acids catalysts with two different methods of fusion and wet-impregnation. XRD results suggested that solid solutions of nano-crystalline SnO_2_ were formed due to the incorporation of Mo and W cations into the SnO_2_ lattice. Textural characterization results revealed that all the compounds showed smaller crystallite size, large specific surface area (WO_3_-SnO_2_ = 32 m^2^/g and MoO_3_-SnO_2_ = 56 m^2^/g), and high porosity. Moreover, Raman measurements and TPR results confirmed the formation of more oxygen vacancy defects in the doped catalysts along with facile reduction of the doped SnO_2_, respectively. The positive impact of Mo and W oxides on the acidic properties of the SnO_2_ was revealed by NH_3_-TPD. Total acidity of MoO_3_-SnO_2_ and WO_3_-SnO_2_ were 81.45 and 61.81 μmol/g, respectively. The presence of larger number of Brønsted (B) acidic sites vs. Lewis (L) sites (B/L = >95%) was confirmed by pyridine-FTIR characterization. High selectivity to solketal (96%) at approximately 70% glycerol conversion was achieved over the MoO_3_-SnO_2_ sample at the optimum reaction condition of 1:1 glycerol to acetone molar ratio, 5 wt% catalyst loading in 150 min. In addition, this catalyst reached around 65% solketal selectivity at almost complete glycerol conversion in the presence of furfural (1:1 glycerol to furfural molar ratio) in 120 min. Finally, applications of different mono-substituted furfural compounds (e.g., 5-methylfurfural, 5-nitrofurfural, 5-chlorofurfural, and 5-hydroxymethyl furfural) were evaluated for acetalization of glycerol in the presence of MoO_3_/SnO_2_ sample. The results confirmed that all the substituted compounds reached lower glycerol conversion than furfural. This observation confirms the impact of steric hindrance induced with substitutes rather than the electronic effects of the substituent (i.e., inductive, resonance and hyper conjugation influences). In detail, the acetalization reaction reached >61% solketal selectivity at >60% glycerol conversion in the presence of different mono-substituted furfural.

In another study, Gonzalez-Arellano et al. (2014b) also evaluated the application of various formaldehyde sources and solventless/solvent-containing systems [Formalin (solvent-less), Para-formaldehyde (water), Para-formaldehyde (solventless)] in the presence of Zr-SBA-16 catalyst with three different Si/Zr ratios (100, 50, and 25) for glycerol acetalization to glycerol formal (GF). Results showed that the Zr–SBA-16(100) sample exhibited 24 and 76% selectivity to the solketal and dioxane, respectively, at 77% glycerol conversion at optimum reaction conditions of 100°C reaction temperature, 1:1 glycerol to para-formaldehyde molar ratio. In addition, the Zr–SBA-16(50) sample could be successfully reused up to five times under identical reaction conditions, without any noticeable decrease in activity. The main reason for better stability of Zr-SBA-16 (50) compared to the Zr-SBA-16 (100) was acidity. Indeed, the Zr-SBA-16(50) possessed higher amount of total acidity (116 μmol/g) with Lewis acidic nature (B/L = 36/80) compared to the Zr-SBA-16 (100) with just 40 μmol/g total acidity and with Bronsted acidic nature (B/L = 28/12).

Gonzalez-Arellano et al. (2014a) continued their study on the acetalization of glycerol with different aldehyde sources (para-formaldehyde, benzaldehyde, furfural, and acetone) in the presence of another newly synthesized heterogeneous catalyst, which was supported iron oxide nano-particle system of a mesoporous alumino-silicate heterogeneous catalyst (Fe/Al-SBA-15). The characterization results confirmed that Fe/Al-SBA-15 possessed high surface area (688 m^2^/g) with Brønsted acidic nature (88 μmol^−1^g^−1^). Experimental results revealed that the product distribution was totally dependent on the use of different aldehyde sources. In fact, acetalization of glycerol with para-formaldehyde results in the production of dioxane (selectivity 66%) as the main product compared to the dioxolane with just 34% selectivity at almost complete glycerol conversion. In contrast, the use of other aldehyde sources led to production of dioxolane (solketal) as the main product. The product's selectivities (dioxolane/dioxane) were 84%/16% at 70% glycerol conversion, 60%/40% at >95% glycerol conversion, and 99%/1% at 58% glycerol conversion in the presence of benzaldehyde, furfural and acetone, respectively. Finally, the Fe/Al-SBA-15 showed the highest stability after five consecutive runs without significant reduction in catalyst activity in the presence of acetone as the aldehyde source.

Gadamsetti et al. (2015) synthesized a series of supported SBA-15 with molybdenum phosphate (MoPO 5–50 wt%) catalysts for the acetalization of glycerol with acetone. Synthesized catalysts were characterized and the XRD results revealed that unsupported MoPO exhibits the formation of (MoO_2_)_2_P_2_O_7_ phase and is dispersed well on the SBA-15 surface. Also, Raman spectra characterization confirmed the existence of MoPO species [(MoO_2_)_2_P_2_O_7_] in samples with more than 40 wt% MoPO supported on SBA-15. In addition the UV-DRS results revealed the presence of both isolated tetrahedrally and isolated octahedrally coordinated Mo centers in the supported and unsupported MoPO. Finally, the NH_3_-TPD analysis shows that the total acidity surged from 0.2 to almost 1 mmol/g with MoPO loading from 5 to 40 wt%; however, total acidity dropped by increasing MoPO loading beyond 40 wt%. In contrast, specific surface area of synthesized catalysts showed a downward trend from 688 to 125 m^2^/g for 5 to 50 wt% of MoPO loading. Acidity of catalysts had negative impact on catalytic performance. The 40 wt% MoPO/SBA-15 sample showed the best catalytic activity with 98% Solketal selectivity at complete conversion (100%) at optimal reaction condition of 3:1 molar ratio of acetone to glycerol, 50 mg catalyst loading, room temperature, in 2 h.

da Silva et al. (2017) used solid SnF2 catalyst for glycerol ketalization with propanone to solketal. The SnF_2_ catalyst reached 97% selectivity of solketal at 97% glycerol conversion at optimum condition of glycerol (21.0 mmol), propanone (168.0 mmol) molar ratio (1:8), CH_3_CN (15 mL), at room temperature (298 K). Most importantly, this catalyst exhibited incredible stability even after four times recycling and reuse with almost constant reaction conversion and solketal selectivity.

Another recent study on a series of zirconia-based catalysts for the acetalization of glycerol suggested that the activity increased in the order of ZrO_2_ < WO_x_/ZrO_2_ < MoO_x_/ZrO_2_ < ${\text{SO}}_{4}^{2-}$/ZrO_2_. In particular, the use of a sulfated zirconia catalyst led to ~98% conversion of glycerol and ~97% selectivity to solketal. The surface acidity and crystalline state of ZrO_2_ on the ${\text{SO}}_{4}^{2-}$/ZrO_2_ catalyst were found to be very influential to the catalytic performances (Reddy et al., 2011).

Kapkowski et al. (2017) synthesized a series of nano-silica supported Re, Ru, Ir, Rh NPs along with different mixture of the metal (Re, Ru, Ir, and Rh) catalysts for acetalization of glycerol with acetone or butanone. It was found that nano-SiO_2_ supported Re (1.0%Re/SiO_2_) was a highly efficient catalyst in glycerol acetalization reaction for solketal production exhibiting the highest activity (TOF = 620.7 h^−1^) with 94.1% selectivity to solketal at 100% glycerol conversion. The addition of Ir (1.0% Re.Ir (1:1)/SiO_2_) could also slightly improve the solketal selectivity to 96% and catalyst activity to TOF = 630.5 h^−1^ at complete conversion. Although 1.0%Re/SiO_2_ favors five-membered cycles, its substitution with Mo alters this selectivity and both five- and six-membered products can be obtained. In detail, the solketal selectivity and catalyst activity decreased to about 78.9% and 336.9 h^−1^, respectively. Despite the addition of Rh [1.0%RuRh(1:1)/Mo], the solketal selectivity did not increase more than 93.4%.

**Figure 7:**
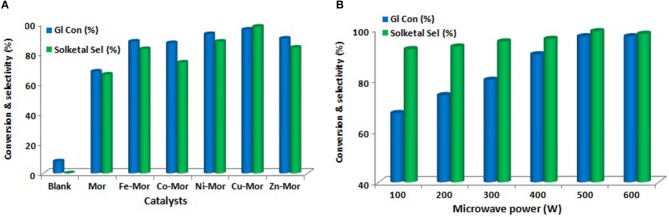
**(A)** Glycerol acetalization using different mordenite catalysts. **(B)** Effect of microwave power on glycerol acetalization over Cu-Mor catalyst.

**Figure 8:**
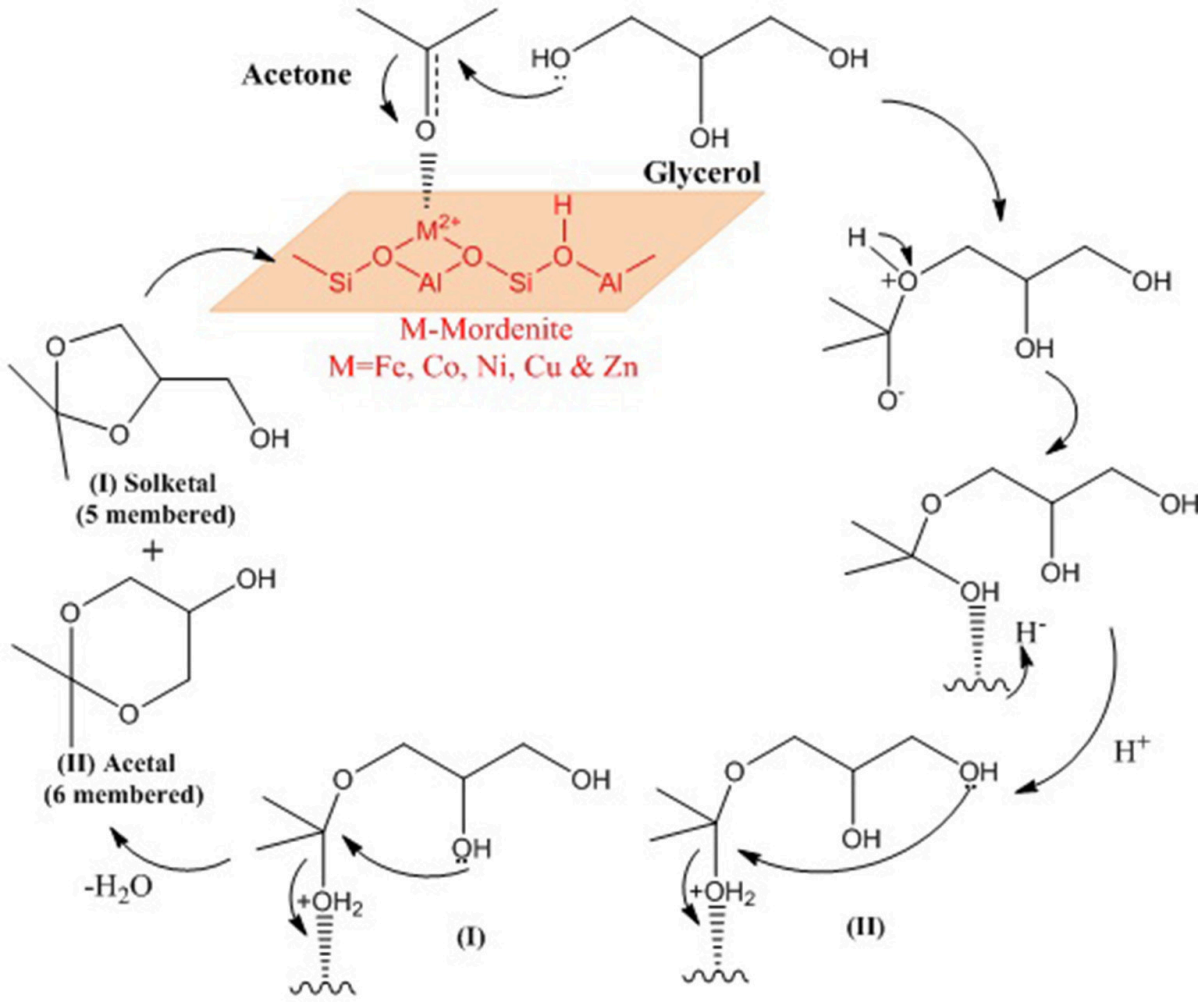
Plausible reaction mechanism of glycerol acetalization over metal promoted mordenite catalysts.

Priya et al. (2017) used microwave irradiation as a heating source in glycerol acetalization to fuel-additives over different transition-metal-ion-promoted mordenite solid acid catalysts which were synthesized by wet impregnation method. The transition metal ions include Fe, Co, Ni, Cu, and Zn. This approach is considered notably clean and green in this field. The results from the microwave irradiation system were compared to those from other processes that use conventional heating sources to ascertain its efficiency and efficacy. The Cu-Mor catalyst showed the highest activity because of the large number of acidic sites and the synergetic effects of metal particles interacting with mordenite. The activity of synthesized samples is shown in Figure 7A. Using Cu-Mor sample and 3:1 acetone/glycerol molar ratio, 98% solketal selectivity at 95% glycerol conversion was obtained in only 15 min. Figure 7B illustrates how the glycerol conversion and solketal selectivity in the presence of the best sample of Cu-Mor varies with microwave power. The reaction mechanism of glycerol acetalization using microwave irradiation and using Cu-Mor catalyst was proposed (Figure 8). Finally, the Cu-Mor sample exhibited an excellent reusability of up to four reaction cycles with only a marginal drop in reaction conversion.

Timofeeva et al. (2017) investigated glycerol acetalization with acetone using iso-structural MOFs of the families MIL-100(M) and MIL-53(M) (M = V, Al, Fe, and Cr) and mixed MIL-53(Al/V). The results revealed that the metal ion's type in MIL-100(M) and MIL-53(M) has significant impact on the rate of reaction and selectivity of desired product. The zero point of charge of the surface (pH_PZC_) values are revealed that the acidity of MIL-100(M) dropped in the following order: MIL-100(V) > MIL−100(Al) > MIL-100(Fe) > MIL-100(Cr). As a result, glycerol conversion decreases in the following order V^3+^ > Al^3+^ > Fe^3+^ > Cr^3+^. Indeed, literature analysis revealed that isomer selectivity depends on the length of the M-O bond in MIL-53(M) and MIL-100(M). Thus, length of M-O bond in MIL-53(M) and MIL-100(M) change in the following order: (Å): MIL-53(Cr) [2.08 (Serre et al., 2002)] > MIL-53(Al) [1.82–2.00 (Loiseau et al., 2004)] > MIL-47(V) [1.946–1.998 (Karin et al., 2004)] and MIL-100(Cr) [2.18 (Férey et al., 2004)] > MIL-100(Fe) [2.065 (Horcajada et al., 2007)] > MIL-100(Al) [1.831–1.995 (Volkringer et al., 2009)], respectively. The decrease in the length of the M-O bond favors increased formation of solketal for both samples. The solketal selectivities increased from approximately 80, 87, and almost 90% over MIL-100(Cr), MIL-100(Fe), and MIL-100(Al). Similarly, it surged from about 80 to 90%, and then around 97% for MIL-53(Cr), MIL-53(Al), and MIL-53(V), respectively. Evaluation of mixed MIL-53(Al,V) showed that the reaction rate and solketal selectivity rise from 90 to 97.5% with increasing V^3+^ content from 0 to 1% in MIL-53(Al,V). Also, the efficiencies of MIL-100(V) (87 mol/mol) and MIL-47(V) (106.1 mol/mol), were higher than those of H_2_SO_4_, SnCl_2_ and p-toluene sulfonic acid with 50.9, 89.6, and 58.9 mol/mol, respectively at 25°C. The MIL-100(V) catalyst exhibited four times recycling and reusability with negligible reduction in glycerol conversion (>80%).

de Carvalho et al. (2017) synthesized a series of titanatenano-tubes (TNTs) by hydrothermal method (sodic and protonic TNTs) to investigate the impact of the type of materials and synthesis time. Physico-chemical characterization results revealed that diversities in the TNT tubular structures with inter wall distances (1.07 to 1.11 nm) depend on the applied synthesis time. TEM, SEM, and XRD characterization results confirmed the enlargement of the layers in the protonic titanates unlike the sodic ones. Sodic TNTs, with long synthesis time of up to 24 h, has the Na_2_Ti_3_O_7_ phase, whereas the protonic TNTs has H_2_Ti_3_O_7_ ones. Although long hydrothermal treatment times (72 h at 160°C) exhibited a strong impact on the reduction of the structural order of the TNTs, it does improve the textural characteristics and acidities of the solids. Also, mild reaction conditions were ineffective for conversion of glycerol over most synthesized sodic TNTs. The best glycerol conversion was obtained over the HTNT sample synthesized at 72 h, with 44.4% glycerol conversion and 83 and 15% selectivity to solketal and acetal, respectively, and only 2% selectivity to by-products (mostly cyclic products) at 50°C and 1:1 acetone to glycerol molar ratio.

Pawar et al. (2015) investigated the glycerol acetalization to fuel-additives using an acid-activated clay catalyst of 6/BBnU/6 in the presence of different ketones (cyclohexanone, Benzaldehyde, Ph-acetaldehyde, Furan aldehyde) in liquid phase. Also, they evaluated the effect of various processes such as solvent-free, conventional thermal activation, and non-conventional microwave/ultrasonic activation methods to find the best operating conditions. Almost complete (99%) selectivity to solketal at 45% conversion was obtained at 1:1 molar ratio of glycerol to cyclohexanone, at room temperature in 3 h. The optimization results revealed that increasing the reaction temperature to 60 °C, glycerol to cyclohexanone molar ratio to 3:1 and reaction time to 20 h could significantly increase the reaction conversion to more than 80%. In addition, cyclohexanone showed the best effect on the acetalization reaction among all the tested ketones. The eco-friendly process involving a catalyst, microwave, or ultra-sonication were successfully utilized to achieve a commercially valuable hyacinth fragrance. About 98% selectivity to solketal could be achieved under microwave and ultrasonic processes at 92 and 89% ketone conversion.

In some cases metal-based catalysts support with different types of carbons [e.g., activated carbon (AC), carbon nano-tubes (CNT), and multi wall carbon nano-tube (MWCNT)] (Khayoon and Hameed, 2013; Khayoon et al., 2014). Carbon materials have been proven to be a good catalytic support in liquid phase reactions due to acid and base resistance, porosity, high surface area, excellent electronic properties, surface chemistry control, and the possibility of metal support (Demirel et al., 2007). For instance, CNT's external surface area led metals to be highly exposed and accessible to reactants, which improves the efficiency. All these spectacular characteristics provide more opportunities for further investigation of carbon materials in glycerol acetalization reaction. Application of other types of metal-based catalysts particularly MOFs in this field were rarely reported. These types of catalysts have showed great results in other research areas such as production of biodiesel (Rafiei et al., 2018), due to their physico-chemical characteristics (e.g., large surface area and porosity), and their applications should be accelerated in the glycerol acetalization reaction.

### Polymer based catalysts

Environmentally friendly chemistry plays an important role in design of a cheap and novel catalyst by utilizing cheaper materials (Kobayashi and Miyamura, 2010). In this regard, different approaches such as micro-encapsulation has been recently recognized as a useful technique to immobilize metal catalysts onto polymers (Akiyama and Kobayashi, 2009). The micro-encapsulation refers to the incorporation of an active substance in a shell or a matrix of a carrier component. The catalysts could be separated from a reaction mixture by simple filtration and recycled, making them suitable for green chemistry processes (Ley et al., 2002). Despite all the recent efforts in this field, the described methods constantly suffers from various issues (e.g., complex systems, tedious processes, difficult control of particles' size and shape, and low activity) (Akiyama and Kobayashi, 2009).

**Figure 9:**
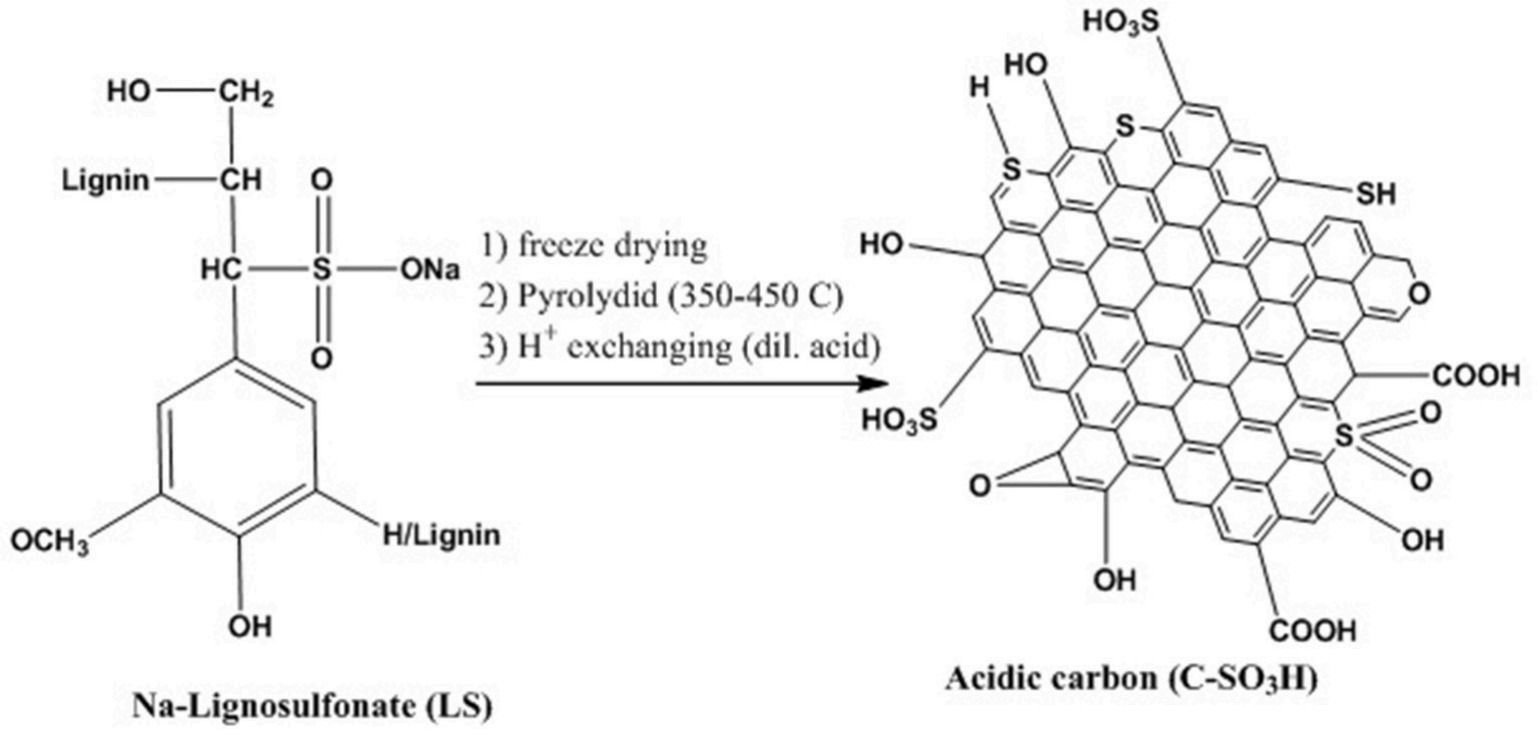
Method for preparing sulfonic acid functionalized carbon materials from LS.

A new and environmentally friendly method was introduced by Konwar et al. (2017) for synthesizing a strong solid acidic meso/macro-porous carbon catalyst from Na-lignosulfonate (LS), which is a byproduct from sulfite pulping. Ice-templated LS was altered to macro/mesoporous solid protonic acid at mild pyrolysis temperature (350–450°C) and through ion/H^+^ exchanging approach (Figure 9). According to the characterization results, the LS-derived components that were synthesized contained heteroatom-doped (O, S) carbon structures that are macro/mesoporous and highly functionalized, as well as a large number of surface -OH, -COOH, and -SO_3_H groups, making them similar to sulfonated carbon materials. In addition, these carbon components exhibited very good activity as solid acid catalysts in glycerol acetalization with various bio-based aldehydes and ketones, easily outperforming the commercial acid exchange resins (Amberlite®IR120 and Amberlyst®70). The highest ≥99.5, 53, and 51% selectivities of solketal were obtained at almost complete glycerol conversion in the presence of acetone, methyl levulinate and furfural, respectively in batch processes. In addition, the optimum LS catalyst (80LS20PS450H^+^) exhibited a large specific surface area (122 m^2^/g) and stable -SO_3_H sites (1.21 mmol/g) revealing excellent potential for continuous production of solketal (reaction condition: 100°C reaction temperature, 60 min time, 50 ml/min N_2_ flow), maintaining its activity (50% selectivity to solketal at ≥91%conversion of glycerol) even after 90 h reaction time.

For the first time, Qing et al. (2017) used a catalytic active membrane synthesized by immersion phase inversion to accelerate the glycerol conversion in an acetalization reaction by continuous removal of water. Indeed, a highly porous “sponge-like” catalytic layer was immobilized with catalyst Zr(SO_4_)_2−_4H_2_O and coated on a polyvinyl alcohol/polyether sulfone per-evaporation membrane. They compared the catalytic activities in batch reactor, catalytically active membrane reactor, and inert membrane reactor. The results revealed the absence of any type of equilibrium limitations for glycerol conversion in the catalytically active membrane reactor and inert membrane reactor. The impact of different operational conditions on synthesis performance in the catalytically active membrane reactor were evaluated, illustrating that higher feed volume (A/V) ratio and temperature enhanced glycerol conversion due to the enhancement of water removal rate. The highest 93% glycerol conversion was achieved at the optimum condition of 5 wt% catalyst concentration, membrane area to A/V ratio of 50/108, 1.2:1 cyclohexanone to glycerol molar ratio, at 75°C in 25 h reaction time.

Organometallic complex, such as cationic oxorhenium (V) oxazoline complex and [2-(2′-hydroxyphenyl)-2-oxazolinato (-2)] oxorhenium (v), were recently tested for a reaction of glycerol with furfural. As a result, solketal selectivity of 70 at 80% glycerol conversion was obtained at 100°C in 4 h (Wegenhart and Abu-Omar, 2010). Also, Crotti et al. (2010) investigated the organo-iridium derivatives [Cp^*^IrCl_2_]_2_ (Cp^*^ = pentamethylcyclopentadienyl) and [Cp^*^Ir(Bu_2_-NHC)Cl_2_], (Bu_2_-NHC = 1,3-di-nbutylimidazolylidene) as the catalysts for the acetalization and transacetalization of glycerol with ketones and aldehydes. Solketal was major product in all those catalytic reactions.

## Impacts of various reaction parameters on catalytic performance

### Reaction temperature

Serafim et al. (2011) evaluated the influence of reaction temperature on the glycerol conversion. It was found that by raising the temperature from 30°C to 70°C, the conversion of the reaction with butanal was greatly improved from 40 to 87%. Improvement was also observed in the selectivity to solketal. With a similar concept, Khayoon and Hameed (2013) reported a moderate increase in reaction temperature led to increase of glycerol conversion and the reaction successfully formed solketal. Reaction between butyraldehyde and glycerol using Amberlyst 47 catalyst and stoichiometric feed ratio in the temperature range of 50–80°C was performed by Guemez et al. (2013). The total reaction rate increased with temperature although this growth did not affect the final equilibrium conversion. The same results were demonstrated for the reaction of glycerol with formaldehyde and acetaldehyde (Agirre et al., 2011).

In contrast with the aforementioned results and statements, some researchers revealed that product selectivity could be reduced with increasing reaction temperature. Nanda et al. (2014a) showed that a higher temperature lowered the product yield for exothermic reactions. More specifically, the increase in reaction temperature only affected the initial reaction rate. In addition to the mentioned studies, Shirani et al. (2014) reported reduction of solketal yield, by increment of the acetone to glycerol molar ratio as well as the temperature. As the temperature increased, the efficiency of the liquid glycerol molecules with the interaction of the gaseous acetone molecules on the catalyst surface decreases, which caused a lower conversion, yield, and solketal production. Indeed, acetone was vaporized and glycerol was still in the liquid phase while temperature was increased. To enhance the quantity of six-membered cyclic acetal in the mixture, a mild reaction temperature was found to be favorable for the catalytic condensation of glycerol with para-formaldehyde using Amberlyst−36 catalyst (Deutsch et al., 2007).

### Application of various reactants

Many studies have confirmed that the reactant design has a significant impact on reaction conversion and product selectivity in the glycerol acetalization reaction. Agirre et al. (2011) examined various mole ratios of glycerol and formaldehyde, using Amberlyst 47 catalyst. The study was performed by altering the molar ratio of glycerol: formaldehyde from 1:1; 1:2 and 1:3, at 353 K. The equilibrium conversion of the formaldehyde increased with rising molar ratio of glycerol. They further applied in excess glycerol for the glycerol and acetaldehyde reaction, which led to 100% conversion of in all cases (Agirre et al., 2013). This finding was in line with Nanda et al. (2014a) on the condensation of glycerol with acetone whereby the glycerol and acetone molar ratio significantly affected the kinetics and thermodynamics of the reaction. When the molar ratios of acetone to glycerol were 1.48:1 and 2.46:1, the solketal yields were 68 and 74%, respectively. The influence of ethanol as a solvent that enhances solubility in acetone was also studied and showed insignificant effect. In another study, the effect of acetone in excess on glycerol conversion was investigated by Ferreira et al. (2010). The glycerol conversion improved with increasing glycerol to acetone molar ratio (from 1:3 to 1:12), while the selectivity to solketal remained constant.

Khayoon and Hameed (2013) stated that in the presence of 5%Ni−1%Zr/AC, the glycerol conversion and the formation of six-membered cyclic ketals were improved by raising the glycerol to acetone molar ratio from 1:4 to 1:8. The same results were reported by Guemez et al. (2013) for glycerol and n-butyraldehyde. When the initial glycerol to butyraldehyde ratio increased from 1:1 to 3:1, the n-butyraldehyde conversion at 80°C and 100 min of reaction time increased from 88 to 98%. When the glycerol to butyraldehyde molar ratio was 0.2, the glycerol conversion reached 100% after 40 min. In another research, the effect of glycerol/butanal molar ratio on the glycerol conversion was investigated (Serafim et al., 2011). In the presence of BEA zeolite catalyst at 80°C, the glycerol conversion after 4 h reaction for glycerol/butanal molar ratio of 1:1 was 71%, whereas the conversion reached 88% in the molar ratio of 1:2.5. However, the further increase of molar ratio (1:6) did not affect the conversion. Applying Amberlyst 15 as a catalyst, Faria et al. (2013) investigated the effect of various solvents on the production of glycerol ethyl acetalin a simulated moving bed reactor via acetalization of glycerol with acetaldehyde. Compared with acetonitrile, and N,N dimethyl formamide, dimethylsulfoxide solvent exhibited better results owing to its capacity toward the catalyst adsorbents, inertness, and miscibility with the reaction medium. da Silva et al. (2015a) used an available, environmentally friendly, efficient and simple tin-based catalyst for the ketalization of glycerol with different ketones at 25°C. The ketones conversions were 40 and 98% for 4-methyl 2 pentanone and cyclohexanone, respectively.

### Water removal

The acetalization reaction is reported to have a low equilibrium constant (Garcia et al., 2008). Thus, shifting the equilibrium to the product (solketal) side would lead to a higher glycerol conversion. This could be executed by removing the water continuously generated during the reaction or by feeding excess acetone into the reactor. However, the former approach is reported to be the more effective method to break the thermodynamic barriers.

Entrainers have been used in different processes for the continuous elimination of the water from a reaction mixture (Ag, 1998). Benzene, chloroform, and petroleum ethers are some of the entrainers that can be used in this process. The effectiveness of these entrainers is not excellent since their boiling points are higher than acetone. Co-distillation of acetone leads to low efficiency in azeotropic water removal. This obstacle was observed when petroleum ether was used as an entrainer (Chen et al., 2005). The application of phosphorous pentoxide and sodium sulfate as catalyst and desiccant to remove water from the reaction environment has also been reported (Ag, 1998). However, the high amount of catalyst consumption in these cases raises operation costs (He et al., 1992). The aforementioned obstacles could be solved by enhancement of acetone utilization, which not only acts as a reactant but also acts as an entrainer. More importantly, the excess acetone could be recycled and reused in the same process, or even other processes.

Roldan et al. (2009) used membrane batch reactor rather than conventional batch reactor to eliminate water from the reaction environment. Also, Vicente et al. (2010) investigated a two-step batch mode operation for continuous removal of water from the reaction environment. The reaction mixture comprising glycerol, acetone and catalyst was stirred under reflux in a 100 mL flask at 70°C (first step), followed by the removal of produced water as well as acetone by vaporization under vacuum at 70°C. Fresh acetone was added to maintain the liquid level to start a new cycle (second step). After three consecutive steps, 90% solketal yield was obtained at the optimum reaction conditions of 70°C reaction temperature, 5 wt% loading of ArSBA- 15 catalyst, and 30 min reaction time for each step.

### Application of different types of processes

Batch reactors commonly encounter drawbacks when scaling up the process. Solketal production in a continuous-flow reactor and in the presence of heterogeneous catalyst is an effective solution because it leads to higher heat and mass transfer efficiency, easy scaling-up of the process along with more environmental and economic advantages (Noël and Buchwald, 2011). Initial attempts were not successful. For example, Clarkson et al. (2001) used a semi-batch reactor while acetone was fed continuously while the glycerol amount was constant. The main obstacle for a continuous process was glycerol's high viscosity particularly at low temperatures. In another attempt, Monbaliu et al. (2011) designed a continuous process using homogeneous H_2_SO_4_ as catalyst. However, application of H_2_SO_4_ made the process not environmentally friendly due to the corrosion and waste disposal problems.

Cablewski et al. (1994) reported on the application of a continuous microwave reactor (CMR) in the production of solketal. A solution of glycerol, acetone and catalyst (pTSA) was pumped into the microwave cavity at a desired temperature, resulting in 84% solketal yield at 13.5 molar ratio of acetone/glycerol, 132°C reaction temperature, 1,175 kPa pressure, 1.2 min residence time and 20 mL/min feed flow rate. However, this process was not appropriate for use with heterogeneous catalysts and low reaction temperatures or for reactants that are incompatible with microwave energy.

**Figure 10:**
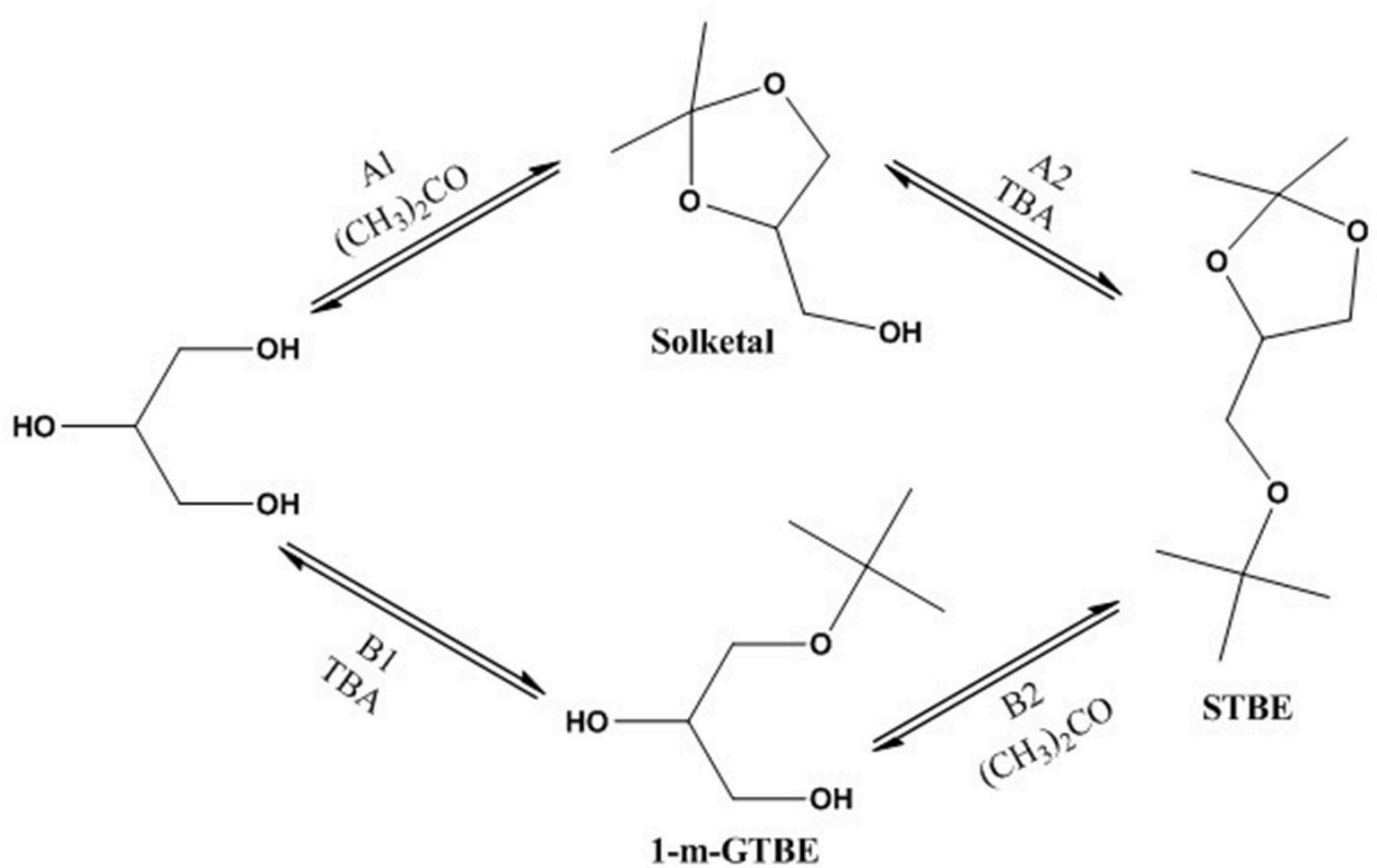
Possible pathways of STBE formation.

For the first time, Samoilov et al. (2016) studied the concurrent ketalisation–alkylation processes in a continuous flow fixed-bed reactor. The results revealed that a continuous single-step process could be effective for glycerol conversion to a mixture of ethers at mild reaction temperature (40–70°C) over a zeolite BEA catalyst. The STBE could be produced at 30% molar yield at optimum reaction conditions of 1:3.4:10 glycerol: TBA: acetone molar ratio and at 45°C. The data in Table 4 show that solketal has significant influence on the anti-wear characteristics. Application of the glycerol derivatives (e.g., 0.046–2.25 wt%) into hydrocarbon oil can enhance the anti-wear characteristics by 42%. The substitution of the solketal hydroxyl group by TBA produces large hydrocarbon radicals, which could slow down the adsorption of the molecule on metal surfaces by steric hindrance along with a change in the polarity and molecules' surface activity. Moreover, the spatial configuration of the ketal molecule may have a positive impact on the adsorption. Figure 10 illustrates the different steps of STBE formation. The first route (A) includes glycerol ketalisation (A1) followed by solketal tert-butylation with TBA (A2). The second route (B) includes formation of 1-mono-GTBE (B1) and then ketalisation of ether to STBE (B2).

**Table 4:** Effect of glycerol ether additives on the antiwear properties of heavy cycle oil (ASTM D 2266-01 test method).

**Run**	**Additivies**		**Average WSD^a^ (mm)**	**ΔWSD^b^ (%)**
	**Type**	**Amount (ppm)**		
1	Additive-free cycle oil		0.94	–
2	Solketal	460	0.71	25
3		980	0.61	35
4		22,470	0.54	43
5	Mixture of di-GTBEs	5,250	0.76	19
6		1,200	0.84	11
7		440	0.88	6
8	STBE/solketal, 70/30	490	0.87	7
9		1,242	0.82	13
10		5,039	0.61	35

a Wear spot diameter;

b *Relative change to additive-free cycle oil WSD*.

Recently, Nanda et al. (2014b) developed a continuous-flow reactor based on the “Novel Process Windows” concept with respect to temperature, pressure and/or reactant concentration to enhance the intrinsic kinetics of the reaction for an optimum yield. Results indicated that they could achieve more than 97% selectivity to solketal at >80% glycerol conversion using different catalysts (H-β zeolite, Amberlyst 36 wet, Amberlyst 35, ZrSO_4_) at 40°C reaction temperature, 600 psi reaction pressure, 6:1 molar ratio of acetone to glycerol in 15 min reaction time. On the other hand, this system suffered from the catalyst clogging the reactor.

**Figure 11:**
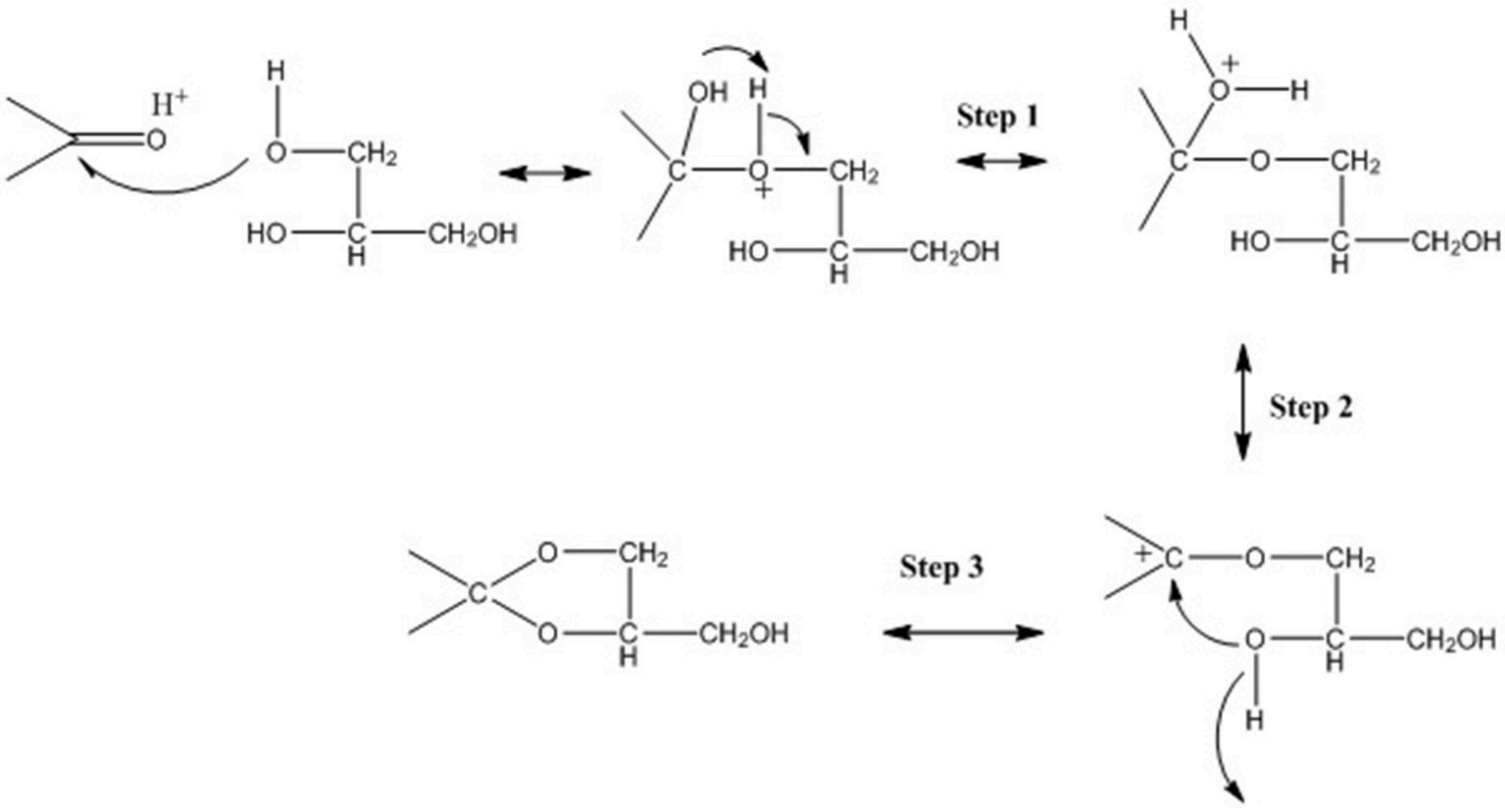
Proposed reaction mechanism for glycerol and acetone acetalization over acid catalyst.

The glycerol acetalization reaction mechanism in the presence of an acid catalyst that leads to the formation of both five- and six-membered rings (ketals) is illustrated in Figure 11 (Voleva et al., 2012). Nanda et al. (2014a) have also reported a two-step processes for glycerol acetalization reaction. The surface reaction between glycerol and the adsorbed acetone on the catalyst surface form the hemi-acetal (Step 1), then the carbonyl carbon turns into a carbocation by the removal of a water molecule and deprotonization to form solketal and 1,3-dioxane. The 1,3-dioxane (six membered ring ketal) is the less favorable product because one of the methyl groups is in the axial position of the chaircon formation (Maksimov et al., 2011). Thus, in the majority of cases the resulting product has higher ratio (even up to 99:1) of five-membered ring (solketal) to six-membered ring (5-hydroxy-2,2-dimethyl-1,3-dioxane).

Perreia and Rodrigues (2013) creatively used process intensification for acetal production along with advanced technologies, applying Simulated Moving Bed Membrane Reactor (PermSMBR), and Simulated Moving Bed Reactor (SMBR), for the acetalization of2-butanol and ethanol with acetaldehyde. These reactors achieved high conversion and productivity at low temperatures (10–50°C), while requiring additional operational costs and capitals. This method could be used for the acetalization of glycerol. The reactive distillation with approximately 100% glycerol conversion is the best reactor design since they feature low capital costs and no reactor clogging with the catalyst. Performing the distillation and reaction simultaneously also saves on operational cost.

Gorji and and Ghaziaskar (2016) synthesized mono-acetin by reacting glycerol with acetic acid (first step) followed by solketal production from the reaction of mono-acetin with acetone over Purolite PD 206 catalyst (second step). This method was reported as an economical and easy to scale up process for glycerol conversion to fuel-additives. Results show 69% yield of solketal was achieved at almost complete glycerol conversion at optimal reaction conditions of 5:1 acetone to mono-acetin molar ratio, 0.2 mL/min feed flow rate, and 2.0 g catalyst at ambient temperature of 20°C and 45 bar pressure in the second step.

### Application of crude glycerol

The key factor for industrial applications of glycerol is its purity level. The crude glycerol obtained from biodiesel production is normally considered as waste by-product in the biodiesel industry since it contains impurities, namely esters, methanol, fatty acids, water, and inorganic salts (matter organic non-glycerol, MONG) (Liang et al., 2010; Rosas et al., 2017). Economically, it is feasible for large-scale biodiesel firms to purify the crude glycerol for further utilizations. Small companies on the other hand cannot afford it; therefore, they have to pay for glycerol disposal or burn it as a waste stream (Wilson, 2002; McCoy, 2006). Heterogeneous catalysts and non-edible oils have been used in the vast majority of studies to produce higher quality biodiesel and glycerol. For example, high quality biodiesel (98.3%) and glycerol (98%) were produced over the Zn-Al heterogeneous solid catalyst (Bournay et al., 2005). Their synthesized catalyst could avoid all the costly purification steps in the direct utilization of crude glycerol. Thus, application of crude-glycerol is another factor that could significantly impact the catalyst activity. Although there have been plenty of studies on the bio-based production of value-added chemicals from refined glycerol, the same cannot be said regarding the utilization of crude glycerol as feedstock.

da Silva and Mota (2011) evaluated the impact of impurities on the formation of solketal in a batch reactor to enable the utilization of crude glycerol rather than purified glycerol. They investigated the effect of common impurities (e.g., 1% methanol, 10% water, and 15% NaCl) in the acetalization of crude glycerol with various heterogeneous catalysts (e.g., H-beta zeolite and Amberlyst-15). When crude glycerol was used in place of refined glycerol, a dramatic drop in reaction conversion was observed, down to 47 and 50% for Amberlyst-15 and H-beta zeolite catalysts, respectively, compared to 95% reaction conversion for refined glycerol with similar catalysts. In fact, they revealed that methanol had less effect than water and NaCl.

Vicente et al. (2010) reported the application of acid-functionalized SBA-15 catalysts for acetalization of crude glycerol (85.8 wt.%). They obtained 81% conversion of glycerol. However, high Na^+^ content in crude glycerol deactivated significantly the sulfonic acid sites due to a cation exchange reaction between Na^+^ and H^+^.

In another study, Nanda (2015) continued their studies by developing a modified continuous-flow reactor including guard reactors that allow online elimination of impurities from the glycerol feedstock and on-line regeneration of deactivated catalysts. A maximum 78% yield of solketal was achieved using crude glycerol and the modified continuous-flow reactor in only 1 h of on-stream time. They also conducted on-line regeneration of the deactivated catalyst in the guard reactor concurrently with the ketalization experiment using purified (96%) crude-glycerol as feedstock. They found that the catalyst (Amberlyst-36 wet) could be effectively regenerated for four consecutive times even up to 96 h of reaction time with only 11% fall (92 to 81%) in solketal yield (Nanda, 2015). For the catalyst regeneration, a 0.5M H_2_SO_4_ solution was passed through the guard reactor, followed by a methanol washing of the regenerated catalyst and finally drying the bed with nitrogen for 5 h.

## Concluding remarks

This review comprehensively summarizes various approaches and strategies for the glycerol conversion to cyclic acetals and ketals processes and recent progress in obtaining higher conversion and selectivity of the desired products. Both homogeneous and heterogeneous acid catalysts can be used in the conversion of glycerol to solketal. The vast majority of studies are conducted over heterogeneous catalysts, which can be easily separated from the system by filtration. Various reaction parameters (e.g., reactor design, temperature, reactants, and nature of catalyst) have significant impact on the catalytic activity in this process.

Recent breakthroughs in catalyst synthesis and characterization lead to unprecedented achievements in this field. Despite various advances, there are still many challenges for increasing selectivity and yield. Indeed, there are spectacular opportunities in catalysis and nano-materials to synthesize a highly active catalyst for glycerol acetalization to specific useful products. Application of more environmentally friendly processes and materials for catalyst synthesis will be the main objective in the future. For instance, application of microwave radiations could enlarge the specific surface area and increased pore volume.

Investigation on the effect of different reaction parameters on catalytic activity revealed that one of the major obstacles in acetalization reaction is its very low equilibrium constant. As a result, the best remedy for this problem is to shift the equilibrium to the product side (e.g., solketal), by either feeding excess reactant (e.g., acetone) or by removing water generated during the reaction. Also, compared to operation in a batch reactor, similar or even higher product selectivity and relatively shorter reaction time could be achieved with the use of continuous flow reactors. Definitely, more investigation on continuous glycerol acetalization process could be one of the major steps toward industrialization and commercialization of this process in the near future.

The reviewed studies confirm that the acetalization of glycerol is a promising process that could bring significant economic prosperity. In addition to the economic benefits, it also has tangible benefits on the environment by shifting the production of industrial chemicals away from petroleum-based toward bio-based processes. Indeed, fuel additive could improve the cleanliness of different parts of the engine, promote complete combustion, reduce fuel gelling and choking of nozzle, as well as reduce corrosion impact on different parts of the engine.

## Author contributions

This research is based on AT-K's postdoctoral fellowship research project. NN assisted in collecting information, the revision process, and manuscript improvement. NA was the corresponding author and AT-K's main supervisor during his Postdoctoral fellowship. ST was AT-K's co-supervisor during his Postdoctoral fellowship.

### Conflict of interest statement

The authors declare that the research was conducted in the absence of any commercial or financial relationships that could be construed as a potential conflict of interest.
